# Characterisation of reproductive tract microbiome and immune biomarkers for bovine genital campylobacteriosis in vaccinated and unvaccinated heifers

**DOI:** 10.3389/fmicb.2024.1404525

**Published:** 2024-08-19

**Authors:** Mst Sogra Banu Juli, Ali Raza, Mehrnush Forutan, Hannah V. Siddle, Geoffry Fordyce, Jarud Muller, Gry B. Boe-Hansen, Ala E. Tabor

**Affiliations:** ^1^Centre for Animal Science, The University of Queensland, Queensland Alliance for Agriculture and Food Innovation (QAAFI), Saint Lucia, QLD, Australia; ^2^Department of Veterinary and Animal Sciences, Faculty of Health and Medical Sciences, University of Copenhagen, Frederiksberg, Denmark; ^3^Department of Agriculture & Fisheries, Charters Towers, QLD, Australia; ^4^School of Veterinary Science, The University of Queensland, Gatton, QLD, Australia; ^5^School of Chemistry and Molecular Biosciences, The University of Queensland, Saint Lucia, QLD, Australia

**Keywords:** biomarker, BGC, cattle, proteomics, reproductive, venereal disease, microbiome

## Abstract

**Background:**

Bovine genital campylobacteriosis (BGC) is a globally important venereal disease of cattle caused by *Campylobacter fetus* subspecies *venerealis*. Diagnosis of BGC is highly challenging due to the lack of accurate diagnostic tests.

**Methods:**

To characterise the biomarkers for *C. fetus venerealis* infection, a total of twelve cycling heifers were selected and categorised as vaccinated (*n* = 6) with Vibrovax® (Zoetis™) and unvaccinated (*n* = 6). All heifers were oestrous synchronised with a double dose of prostaglandin (PGF2α) 11 days apart and when in oestrous intravaginally challenged with 2.7 x 10^9^ CFU live *C. fetus venerealis*. DNA extracted from vaginal mucus samples was screened using a *C. fetus* qPCR and 16S rRNA was characterised using Illumina sequencing (V5-V8 region). Relative abundances of serum proteins were calculated using sequential window acquisition of all theoretical fragment ion spectra coupled to tandem mass spectrometry (SWATH-MS) for all heifers at three timepoints: pre-challenge, post-challenge and post-recovery.

**Results:**

In 16S rRNA sequencing of vaginal mucus, *Campylobacter* spp. appeared two days following challenge in unvaccinated compared to 14 days in vaccinated animals, consistent with the qPCR results. Increased relative abundances of Firmicutes and Campylobacterota were identified after *C. fetus venerealis* challenge and were associated with *C. fetus venerealis* in vaccinated and unvaccinated heifers. Greater relative abundance of *Streptococcus* spp. was observed during oestrous rather than dioestrous. In both vaccinated and unvaccinated heifers, *Acinetobacter* spp. increased after challenge with higher abundance of *Corynebacterium* spp. in the vaccinated group. A total of 130 unique proteins were identified in SWATH analysis of the serum samples, and the number of differentially abundant proteins found was higher in the vaccinated group after recovery from infection compared to pre-and post-challenge (adjusted *P* < 0.05 and Log2FC > 0.2).

**Conclusion:**

Coglutinin, clusterin, HP homologs, vitamin D binding protein and fetuin B were identified as potential biomarkers for *C. fetus venerealis* infection and need further study to validate their efficiency as immune biomarkers for BGC.

## 1 Introduction

Bovine genital campylobacteriosis (BGC) is a major reproductive disease of cattle, distributed worldwide and causing venereal disease following transmission from bulls to cows and *vice-versa* during natural breeding. BGC can reduce the pregnancy rate by 20%, can increase abortions by 10% annually, and is listed as a notifiable disease by the World Animal Health Organization (Mshelia et al., 2010; Michi et al., 2016; OIE, 2021; World Organisation of Animal Health, 2021). In Australia, BGC is ranked 8th for diseases having financial impacts on the beef industry, causing $AUD 43.7 million in losses *per annum* (Shephard et al., 2022). In Brazil, the prevalence of BGC is 68%, and in Argentina, it is the main cause of abortion in cattle (Cantón et al., 2022; Siqueira et al., 2023).

The causative agent of BGC is *Campylobacter fetus* subsp. *venerealis* which is a gram-negative slow-growing micro-aerophilic bacterium. *C. fetus* subsp. *venerealis* is closely related to another subspecies of *C. fetus* called *C. fetus* subsp. *fetus*, which normally inhabits the bovine intestinal tract and is responsible for sporadic abortion in cattle. It is phenotypically and genetically very similar to *C. fetus* subsp. *venerealis* (Bondurant, 2005; García et al., 2023). The biochemical similarities between the two subspecies make differentiating them difficult, causing false positive results while diagnosing BGC. Although culture is the gold-standard method and can differentiate the subspecies based on biochemical properties, it is challenging due to the slow and fastidious growth of *Campylobacter* subspecies, sensitivity to environmental temperature, and robust growth of other contaminating bacteria (Guerra et al., 2014; García et al., 2023). Despite the development of several serological and molecular diagnostic tests, definitive confirmation of the pathogen by distinguishing the subspecies has not yet been achieved (Van Der Graaf-Van Bloois et al., 2013; Silva et al., 2020; García et al., 2024).

Bulls are asymptomatic carriers of the disease, but symptoms in infected cows are abortion, infertility, and early embryonic death. Cows and heifers produce mucosal immunity after infection, hindering the growth and penetration of the organism into the uterus. Females can expel the bacteria 2–4 months after infection and can regain fertility within 5 months after elimination (Corbeil et al., 1981). Although the use of commercial vaccines is recommended, producers only vaccinate bulls and not females due to high costs (OIE, 2021). Omics applications could assist to further characterise infection with *C. fetus* subsp. *venerealis* without differentiating the subspecies and may identify potential biomarkers for BGC immunity in cattle. Our hypothesis is that through quantitative proteomics and vaginal microbiome sequencing, we can identify biomarkers for BGC which could be developed into valuable predictive, diagnostic, and/or prognostic tools for producers.

Proteomics plays a crucial role in identifying biomarkers for disease in agriculture by providing comprehensive analysis of the proteins involved with cellular and molecular changes at different stages of reproductive diseases. In the last decade, the use of mass spectrometry (MS)-based proteomics such as SWATH-MS and MALDI-TOF-MS has led to the discovery of potential protein biomarkers for several complex diseases in cattle including tick resistance, bovine tuberculosis, and mastitis (Gao et al., 2019; Alnakip et al., 2020; Raza et al., 2021; Birhanu, 2023). More specifically, proteomics techniques have been used to identify host immune biomarkers for endometritis, metritis, cervicitis, vaginitis, retained placenta, purulent vaginal discharge, and repeat breeding syndrome (Miller et al., 2019; Paiano et al., 2022).

The reproductive tract microbiome is a key indicator of reproductive health that can have a crucial role in the development of reproductive tract innate immunity and susceptibility to pathogens or disease. A diverse and balanced microbiome can contribute to the competitive exclusion of pathogenic bacteria and prevent infection by hindering their colonisation of the reproductive tract mucosa (Pinedo et al., 2013). Dysregulation of the microbiome can lead to chronic inflammation, which may negatively impact reproductive tract innate immune processes (Belkaid and Hand, 2014; Sheldon et al., 2014). Most studies of the reproductive tract microbiome of cattle have been undertaken using amplicon sequencing of 16S rRNA (Sicsic et al., 2018; Wang et al., 2018b; Kudo et al., 2021). Proteobacteria, Firmicutes, Fusobacteria, Bacteroidota, and Tenericutes are the most dominant phyla of the bovine reproductive tract microbiome, and their relative abundances alter depending on the reproductive disease and stage of disease progression (Ong et al., 2021). The presence or absence of certain bacterial phyla may serve as biomarkers for both optimal and suboptimal reproduction status and as indicators for reproductive health and therapeutic interventions (Deng et al., 2019). Endometritis, metritis, vaginitis, and cervicitis are the most important inflammatory reproductive diseases of cattle associated with population changes in these bacterial phyla (Jeon et al., 2015; Bicalho et al., 2017). An increased abundance of Fusobacteria and Bacteroidota is found in diseased states, while Firmicutes, Proteobacteria, and Tenericutes are highly abundant in healthy cows (Santos and Bicalho, 2012; Galvão et al., 2019; Moreno et al., 2022).

The advancement of high-throughput analyses has introduced a transformative era in biological research, offering unparalleled methods of understanding pathways of complex diseases and discovering potential biomarkers. In this study, we aimed to understand BGC immunity by using quantitative proteomics to profile serum proteins and to characterise microbiome changes in the reproductive tract of heifers.

## 2 Materials and methods

### 2.1 Source of cattle and ethics statement

A total of 12 cycling Droughtmaster heifers, aged approximately 18–24 months with body condition scores ranging from 3 to 4, were selected for this trial. All heifers were in cycle, and this was confirmed by ultrasound scanning for the presence of a *corpora luteum* (CL). The heifers were sourced from Spyglass Beef Research Facility, Charters Towers, Queensland, Australia, in 2021 and managed as a single group in a paddock at Columba Catholic College (CCC) in Charters Towers (−20° 12′ 33.372” N and 145° 48′ 59.22 E) in North Queensland. Animal Ethics Approval 2021/AE000056 was granted by the Production and Companion Animal (PCA), University of Queensland Animal Ethics Committee.

### 2.2 Vaccine challenge trial

**Figure 1:**
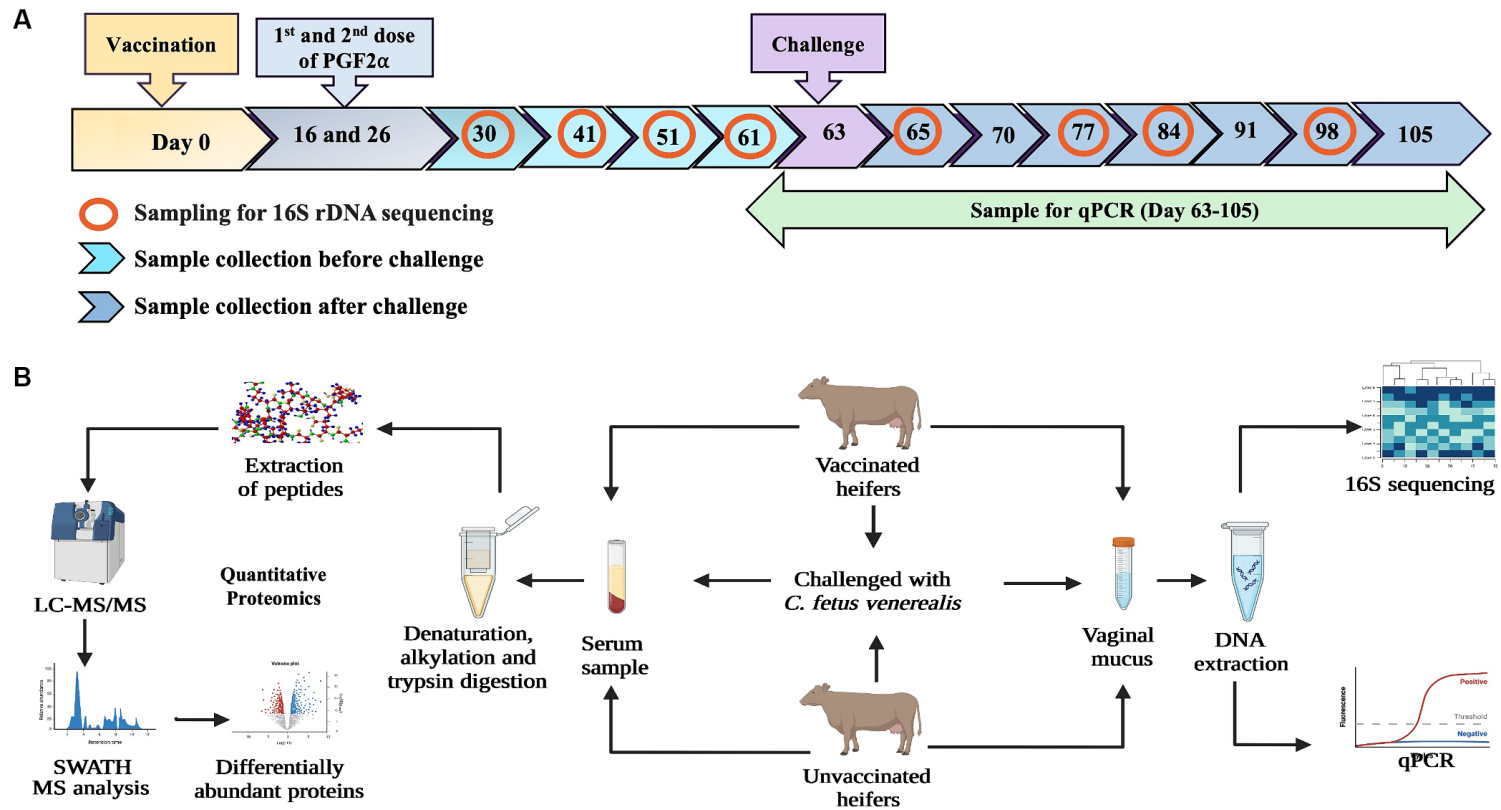
**(A)** Diagram illustrating the timepoints of vaccination, challenge with *C. fetus venerealis* and sample collection. Vaginal mucus samples from vaccinated and unvaccinated heifers were collected at four timepoints before challenge and four timepoints after challenge (marked with red circles) used for 16S rRNA sequencing. Blood samples collected at day-63 (before challenge), day-65 (after challenge) and day-105 (after recovery) were used for quantitative proteomics. **(B)** Workflow of methodology used for immune biomarkers using 16S rRNA sequencing and quantitative proteomics (Created with BioRender.com).

Six heifers were vaccinated once before oestrous synchronisation using a commercially available vaccine against BGC (Vibrovax^®^, Zoetis) following the manufacturer’s instruction, and six were unvaccinated (controls). Nine weeks post-vaccination, both groups were injected with double doses of prostaglandin (PGF2α, Lutalyse^®^; Zoetis) at 11 days apart, to ensure heifers were in oestrus for the intravaginal challenge with 3 mL of freshly prepared inoculum of live *C. fetus venerealis* strain 76223 (Indjein, 2013) bacteria delivering 2.7 × 10^9^ CFU/heifer. The time points for sample collection and the workflow of the vaccine challenge trial are presented in Figure 1.

### 2.3 Preparation of *C. fetus venerealis* inoculum

Freshly grown *C. fetus* subsp. *venerealis* bacterial colonies were collected with disposable cotton tips and mixed in 45 mL of phosphate-buffered solution (PBS). The bacterial suspension was diluted and aliquoted to match turbidity of the McFarland standard of 3, corresponding to 9 × 10^8^ CFU/mL. To confirm the CFU/mL count, 10-fold dilutions of the adjusted inoculum was prepared, and 0.1 mL of each dilution was spread onto 5% sheep blood agar plates using a plastic disposable spreader. For each dilution, two blood agar plates were used. The plates were incubated in an AnaeroJar^™^ (Thermo Scientific^™^, Oxoid^™^) at 37°C for 72 h in a micro-aerophilic atmosphere using a CampyGen^™^ 2.5 L Sachet (Oxoid^™^), and the viable count was determined.

### 2.4 Assessment of heifers and sample collection

Heifers were assessed once pre-vaccination, twice each week for 9 weeks post-vaccination and then weekly for 6 weeks post-inoculation. Assessments included ovarian ultrasound scanning using a 10 MHz rectal probe transducer (Honda 2,100 V, Honda Electronics, Toyohashi City, Japan) to monitor the stage of the oestrous cycle through recording the presence and size of CLs and large follicles. The oestrous cycle of heifers was confirmed at four time points before challenge and four time points after challenge with reference to Prostaglandin injection and ultrasonography scanning. Heifers were weighed at sample collection, and body condition was assessed (1–5 scale) as described previously (Edmonson et al., 1989). At each time point, vaginal mucus was collected using a Tricamper^™^ (Queensland Department of Agriculture & Fisheries, Biosecurity Sciences Laboratory) by inserting the device into the vagina with the leading edge in contact with the dorsal wall of the vagina and moved back and forth in the vagina. After collection, the Tricamper^™^ was transferred into 5 mL of chilled PBS. Coccygeal vein blood samples were collected in 8.5 mL Serum SST-II Advance tubes (BD Vacutainer^®^) at each time point, allowed to clot, and then chilled overnight. Sera were collected following centrifugation at 1900 × g for 10 min, aliquoted, and stored frozen (−20°C) for subsequent progesterone assay and proteomics analyses.

### 2.5 Progesterone assay

Serum samples from vaccinated and unvaccinated heifers were pooled separately, except for heifers in dioestrus before challenge. A competitive Bovine Progesterone ELISA (Colorimetric, Novus Biologicals^™^) quantified the progesterone (P4) concentration in each sample following the manufacturer’s protocol. In brief, thawed serum samples were mixed thoroughly, pooled (within group and time point), and diluted (10-fold) with 0.05% Tween Buffer. Diluted serum samples were added to a 96-well plate with HRP conjugates and antibodies. The reaction was stopped after adding substrates A and B and then read in a Multiskan^™^ FC Microplate Photometer (Thermo Fisher Scientific^®^) at 450 nm. The ELISA was conducted in duplicate with standards and blank samples. A standard curve was generated using four parameter logistic (4-PL) curve fit, and P4 concentrations were detected using linear regression analysis in Graph Pad Prism (version 9.5.1).

### 2.6 Vaginal mucus sample collection, DNA extraction, and sequencing

Vaginal mucus samples were collected four time points before challenge and four time points after challenge using the Tricamper^™^ sampling tool in PBS. After removal of the tool head, the mucus samples were centrifuged at 800 × g to remove the host pellet, and the collected supernatant was centrifuged at 4000 × g to obtain the microbial pellet for DNA extraction. DNA was extracted using the QIAamp^®^ DNA Microbiome Kit (QIAGEN^™^) following the manufacturer’s protocol. In brief, the microbial pellet was subjected to lysis buffer, benzonase enzyme (to deplete host DNA), and bead beating (to lyse bacteria) before column-based purification. The purified DNA was eluted in AVE buffer. The concentration of DNA was measured using the Qubit Fluorometer (Thermo Fisher Scientific^®^), and extracted DNA samples were used for (1) *C. fetus* qPCR and (2) 16S rRNA gene amplicon sequencing through the University of Queensland’s Australian Centre for Ecogenomics (ACE). DNA was amplified by PCR with primers targeting V5–V8 hypervariable regions of the 16S rRNA genes using 803F (5′-TTAGAKACCCBNGTAGTC-3′) and 1392wR (5′-ACGGGCGGTGWGTRC-3′) primers (Engelbrektson et al., 2010), modified to contain Illumina specific adapter sequence (803F: 5′-TCGTCGGCAGCGTCAGATGTGTATAAGAGACAGTTAGAKACCCBNGTAGTC-3′ and 1392wR: 5′-GTCTCGTGGGCTCGGAGATGTGTATAAGAGACAGACGGGCGGTGWGTRC-3′) in NEBNext^®^ Ultra^™^ II Q5^®^ Mastermix (New England Biolabs).

#### 2.6.1 Illumina library preparation for 16S rRNA analysis

Preparation of the 16S rRNA libraries was performed by the Australian Centre for Ecogenomics (ACE), UQ, according to the workflow outlined by Illumina (Illumina, Catalogue No. 15044223). In brief, PCR products of ~580 bp were amplified according to the specified workflow with an alteration in polymerase used to substitute NEBNext^®^ Ultra^™^ II Q5^®^ Mastermix (New England Biolabs #M0544) in standard PCR conditions. The resulting PCR amplicons were purified using Agencourt AMPure XP beads (Beckman Coulter). The purified DNA was indexed with unique 8 bp barcodes using the Illumina Nextera XT 384 sample Index Kit A-D (Illumina FC-131-1002) in standard PCR conditions with NEBNext^®^ Ultra^™^ II Q5^®^ Mastermix. Indexed amplicons were pooled together in equimolar concentrations and sequenced on a MiSeq Sequencing System (Illumina) using paired-end sequencing with V3 300 bp chemistry according to the manufacturer’s protocol. Quality control of the resulting sequences required a minimum of 10,000 raw reads per sample prior to data processing. In addition, the sequences had to meet Illumina’s supplied reagent metrics, achieving an overall Q30 score of greater than 70% for 600 bp reads.

#### 2.6.2 Bioinformatics analysis of sequencing data

Primer sequences were removed from forward de-multiplexed reads using Cutadapt (version 2.10) (Martin, 2011), with reads not containing primers being discarded (--discard-untrimmed). Poor-quality reads were identified and removed with Trimmomatic (version 0.39) (Bolger et al., 2014) using a sliding window of four bases with an average quality threshold of 15 (SLIDINGWINDOW:4:15). Reads were then trimmed to 250 bp (CROP:250), with any less than 250 bp in length discarded (MINLEN:250).

Quality-controlled forward reads were processed using QIIME2 (version 2020.11.1) (Bolyen et al., 2019) for feature selection, abundance calculations, and taxonomy assignment. Reads were de-noised (filtered, dereplicated and chimeras identified and removed) using DADA2 (--p-trunc-len = 0) (Callahan et al., 2016), and relative frequencies of each resulting amplicon sequence variants (ASVs) were calculated. The taxonomy for each ASV was assigned by BLAST analysis of the sequences against a non-redundant 16S SILVA database (release 138, clustered at 99% identity) (Quast et al., 2013) using the classify-consensus-blast function with default parameters. Data were visualised as a box plot, stacked bar, and ordinary plot with *ggplot2*, *ggnewscale*, and *vegan* function (version 2.4-5), and heatmaps were created using *ComplexHeatmap* in R software package version 4.3.1. (Gu et al., 2016; Wickham, 2016). Shannon diversity index was calculated to assess the diversity of a microbiome using *phyloseq*, and beta diversity was calculated using *Bray–Curtis dissimilarity* in R (version 4.2.2).

### 2.7 qPCR

An SNP that differentiated *C. fetus venerealis* and *C. fetus fetus* isolates was identified in the *mraY* gene (Ong, 2022). An “in house” TaqMan^™^ SNP Genotyping Assay (Thermo Fisher Scientific^®^) was developed by labelling *C. fetus venerealis* and *C. fetus fetus*-specific probes with VIC^™^ and FAM^™^ reporter dyes, respectively (Tabor et al., 2024). The AgPath-ID^™^ One-Step RT-PCR Reagent (Thermo Fisher Scientific^®^) without the Reverse Transcriptase step was provided as a 2x concentrated buffer used as the qPCR mastermix. Each reaction consisted of 1x buffer (5 μL), 900 nM of the forward (5’ AAAATGATGATGAATTGGCGCCATT 3′) and reverse (5’ TGTGATGGAAACCTTATCTGTTATATTGCA 3′) primers, 200 nM of the *C. fetus venerealis mraY* VIC probe (5’VIC-CGTTTTTTGTGTATTTT 3’MGBNFQ), 200 nM of the *C. fetus fetus mraY* FAM probe: (5’FAM-CGTTTTTTGCGTATTTT-3’MGBNFQ), and 25X RT-PCR Enzyme Mix (AmpliTaq Gold^™^ DNA Polymerase at 0.025 units per reaction). Assays were run in duplicate in the Bio-Rad CFX96 Touch^™^ Real-Time PCR Detection System for the two fluorophores under the following conditions: activation at 95°C 10 min, followed by 45 cycles of 95°C 15 s, 69°C 1 min, and a final extension at 69°C for 7 min. Raw amplification data (Cq values and relative fluorescence units (RFU)) were exported for analysis in Excel and RStudio (Tabor et al., 2024).

### 2.8 Sample processing for mass spectrometry

Serum samples from five vaccinated (V) and five unvaccinated (UV) cycling heifers were selected for proteomics study based on their qPCR results (*C. fetus* subsp. *venerealis* positivity) at three different time points: (1) pre-challenge (30.06.2021) when all the heifers were negative for *C. fetus venerealis* in qPCR; (2) post-challenge (02.07.2021) when all unvaccinated heifers were qPCR positive and vaccinated heifers were negative, and (3) post-recovery 42 days post-challenge (04.08.2021) when both vaccinated and unvaccinated heifers were qPCR negative. One unvaccinated and one vaccinated heifer showing persistent anoestrus were excluded from the study. The serum proteomes were compared pre-challenge (UV0 vs. V0), post-challenge (UV1 vs. V1), and post-recovery (UV2 vs. V2) to characterise the immune response of heifers with and without *C. fetus venerealis* (see Table 1).

**Table 1:** Grouping of heifers for SWATH-MS analysis across time points with vaccination and oestrous status.

Groups	Time point	Oestrous stage	Vaccination status	
			Unvaccinated (*n* = 5)	Vaccinated (*n* = 5)
Pre-challenge UV0 vs. V0	After vaccination and before challenge with *C. fetus venerealis*	Dioestrous	Unvaccinated (UV0)	Vaccinated (V0)
Post-challenge UV1 vs. V1	After challenge with *C. fetus venerealis*	Oestrous	Unvaccinated (UV1)	Vaccinated (V1)
Post-recovery UV2 vs. V2	After recovery from *C. fetus venerealis* infection	Dioestrous	Unvaccinated (UV2)	Vaccinated (V2)

#### 2.8.1 Sample preparation for SWATH analysis and mass spectrometry

Protein concentration was measured using a Qubit Fluorometer (Thermo Fisher Scientific^®^) using the Qubit Protein Assay kit (Thermo Fisher Scientific^®^). For each sample, 100 μg of total protein was used for further processing using the Pierce concentrator 10 K MWCO columns following the method by Wiśniewski et al. (2009) and Raza et al. (2021). In brief, each sample was incubated at 37°C with 20 × g shaking for 10 min to denature proteins using 8 M urea and 50 mM ammonium bicarbonate (ABC). Denatured proteins were transferred to 10 K MWCO columns for further processing and centrifugation. Two washes were performed with wash solution (8 M urea and 50 mM ABC), and eluted filtrates were discarded after each centrifugation. For the reduction and alkylation of proteins, 5 mM DL-Dithiothreitol (DTT) and 1 M Iodoacetamide (IAA) were used, respectively. Finally, digestion was carried out overnight at 37°C with Sequence Grade Modified Trypsin (Promega), and digested peptides were desalted using C18 Zip Tips (Millipore^®^) following the manufacturer’s instructions. To fractionate, 5 μL of digested peptides were collected from each sample as a pool and fractionated using Pierce High pH Reversed-phase Peptide Fractionation kit (Thermo Fisher Scientific^®^) following the manufacturer’s protocol. Eluted peptides were lipholysed and resuspended in 0.1% trifluoroacetic acid and submitted for SWATH analysis to the School of Chemistry and Molecular Bioscience’s Mass Spectrometry: Proteomics Facility at the University of Queensland.

For the analysis of peptides, LC–MS/MS was conducted using a Shimadzu^®^ Prominence nanoLC system coupled with a TripleTOF 5,600 mass spectrometer equipped with a Nanospray III interface (SCIEX^®^), following previously established protocols (Xu et al., 2015; Raza et al., 2021). In brief, peptides underwent desalting on an Agilent C18 trap (300 Å pore size, 5 μm particle size, 0.3 mm i.d. Å ~ 5 mm) at a flow rate of 30 μL/min for 3 min. Subsequently, separation occurred on a Vydac EVEREST reverse-phase C18 HPLC column (300 Å pore size, 5 μm particle size, 150 μm i.d. Å ~ 150 mm) at a flow rate of 1 μL/min. During separation, a gradient of buffer A (1% acetonitrile/0.1% formic acid) and buffer B (80% acetonitrile/0.1% formic acid) was applied, ranging from 10 to 60% buffer B over 45 min. Gas and voltage settings were adjusted as necessary. MS-TOF scans were performed across the 350–1800 m/z range for 0.5 s in data-dependent acquisition (DDA), followed by DDA MS/MS. The top 20 peptides with intensity greater than 100 were automatically selected across 40–1800 m/z (0.05 s per spectrum) using a collision energy of 40 ± 15 V. For data-independent acquisition (DIA) SWATH analyses, initial MS scans covering 350–1800 m/z were conducted for 0.05 s, followed by high-sensitivity DIA mode. This mode utilised 26 m/z isolation windows for 0.1 s across 400–1,250 m/z. Collision energy values for SWATH samples were automatically assigned by Analyst software based on m/z mass windows (SCIEX^®^).

### 2.9 Data analysis

Data-dependent acquisition (DDA) results were processed using ProteinPilot software (SCIEX^®^ 5.02) and searched against all bovine proteins in UniProtKB (downloaded on 22 February 2022; 47,124 total entries) with settings described by Raza et al. (2021). False discovery rate (FDR) analysis was conducted with limits of 99% confidence and 1% local FDR. To quantify the abundance of peptide in each sample, peak-view analysis was performed using PeakView 2.1 (SCIEX^®^) with specific settings, including allowing shared peptides, a peptide confidence threshold of 99%, FDR set to 1%, XIC extraction window of 6 min, and XIC width of 75 ppm. A linear mixed model using MSstats (version 2.4) in R was conducted to identify differentially abundant proteins with Benjamini and Hochberg corrections to adjust for multiple comparisons and a significance threshold of *p* < 0.05 (adjusted *p*-value) (Kerr et al., 2019). Before conducting the statistical analysis, a Python script was used to remove data that did not pass FDR (significance threshold). The MSstats package was used for normalisation, data verification, and statistical testing to identify differentially abundant proteins. MSstats default setting was used as follows: “equalizeMedians” (default) which represents constant normalisation (equalising the medians) based on reference signals is performed. Variables included in the linear model were the Protein Name, Peptide Sequence, Precursor Charge, Fragment Ion, Product Charge, Isotope Label Type, Condition, Bio Replicate, Run, and Intensity (Choi et al., 2014). All significant proteins were included in subsequent analyses.

Search Tool for the Retrieval of Interacting Genes/Proteins (STRING) was used to identify protein–protein interaction and characterisation for gene ontology (GO) terms for biological processes (BPs) and Kyoto Encyclopedia Genes and Genomes Pathways (KEGG) using Uniprot accession identifiers of significantly differentially abundant (DA) proteins as a target list (Szklarczyk et al., 2019). The *Bos taurus* genome was used as background in the STRING analysis with the following basic settings: meaning of network edges as evidence; active interaction sources included were text trimming, experiments, databases, co-expression, neighbourhood, gene expression, and co-occurrence; high confidence (0.700) for the minimum required interaction score, and *k*-means clustering with the number of clusters set at 3. Volcano plots were made with differentially abundant proteins (adjusted *p* < 0.05 and log2FC > 0.2) using *ggplot2* and *tidyverse* in R (version 4.1.1). Functional enrichment analysis was performed with *clusterProfiller* (version 4.0), and graphs were produced with dot plot and category network plot functions from this package in RStudio.

## 3 Results

### 3.1 Follicular dynamics

As cycling can influence the microbiome content, oestrous and dioestrous phases were confirmed by rectal palpation, ultrasonography, and the progesterone ELISA assay. Follicular dynamics of each individual heifer and progesterone concentration are provided in Supplementary Figure S1.1: Follicular dynamics; Supplementary Figure S1.2: Progesterone (P4) ELISA curve; and Supplementary Table S1.1: Concentration of P4 in samples. Three vaccinated (200,639, 200,668, and 200,681) and one unvaccinated (200578) heifer did not express oestrous synchrony before challenge, while after challenge all heifers followed the expected pattern except for one unvaccinated heifer (200578) which was found consistently in anoestrus and was excluded from further analyses.

### 3.2 Vaccine challenge trial

**Figure 2:**
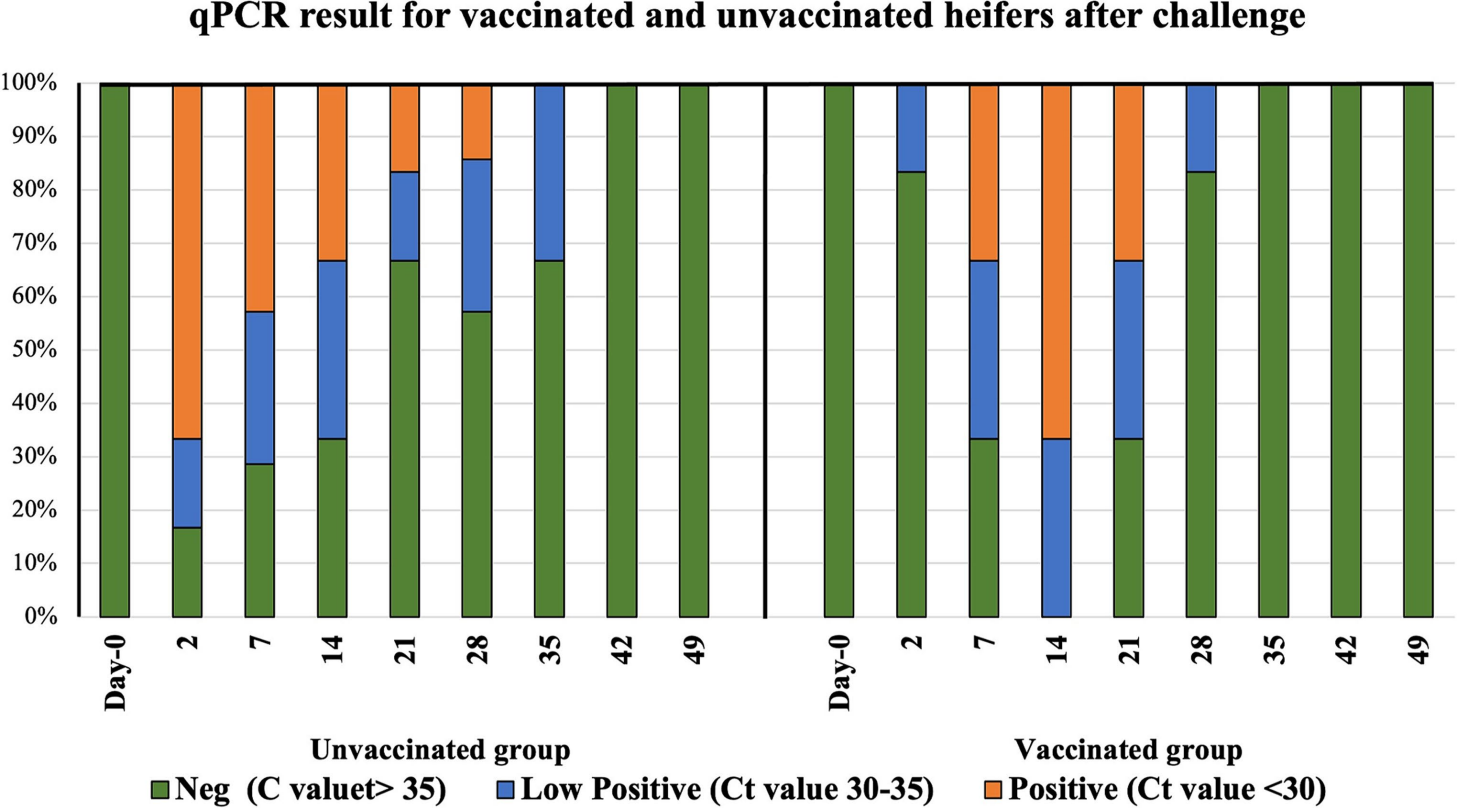
Distribution of infection in vaccinated and unvaccinated heifers by mraY gene qPCR. Day 0 represents sample collection before challenge, and Day 2 to Day 49 are the time points of sample collection after challenge with *Campylobacter fetus* subsp. *venerealis* (strain 76223).

Successful challenge with *C. fetus venerealis* was confirmed by qPCR. Prior to challenge, all heifers were *C. fetus venerealis* qPCR negative at days 2, 7, 14, 21, and 28. After challenge, 67, 50, 33, 17, and 17% of unvaccinated heifers were qPCR positive, compared to 0, 33, 67, 33, and 0% of vaccinated heifers. Vaccinated heifers eliminated the infection sooner than the unvaccinated heifers at 4 weeks versus 5 weeks, respectively (Figure 2 and Supplementary material S2).

### 3.3 16S rRNA microbiome analysis and biomarker identification

#### 3.3.1 Diversity of vaginal microbiome

**Figure 3:**
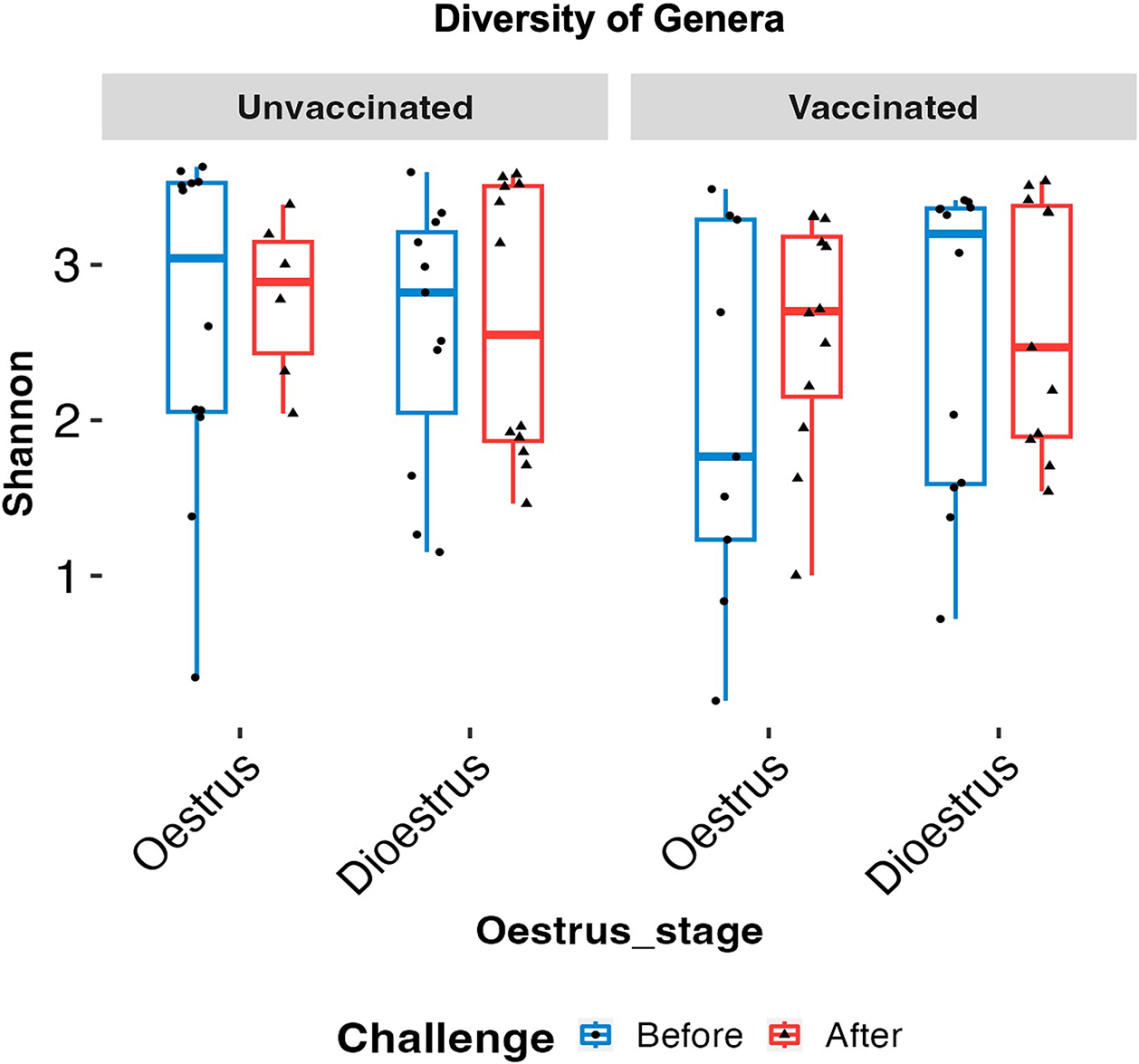
Box plots comparing the Shannon alpha diversity of the genus in oestrus and dioestrus of vaccinated and unvaccinated heifers before and after challenge. Diversity of microbiome was higher in oestrus than dioestrus before challenge in both the vaccinated and unvaccinated group and in dioestrus after challenge. Blue box denotes before challenge and red box after challenge.

**Figure 4:**
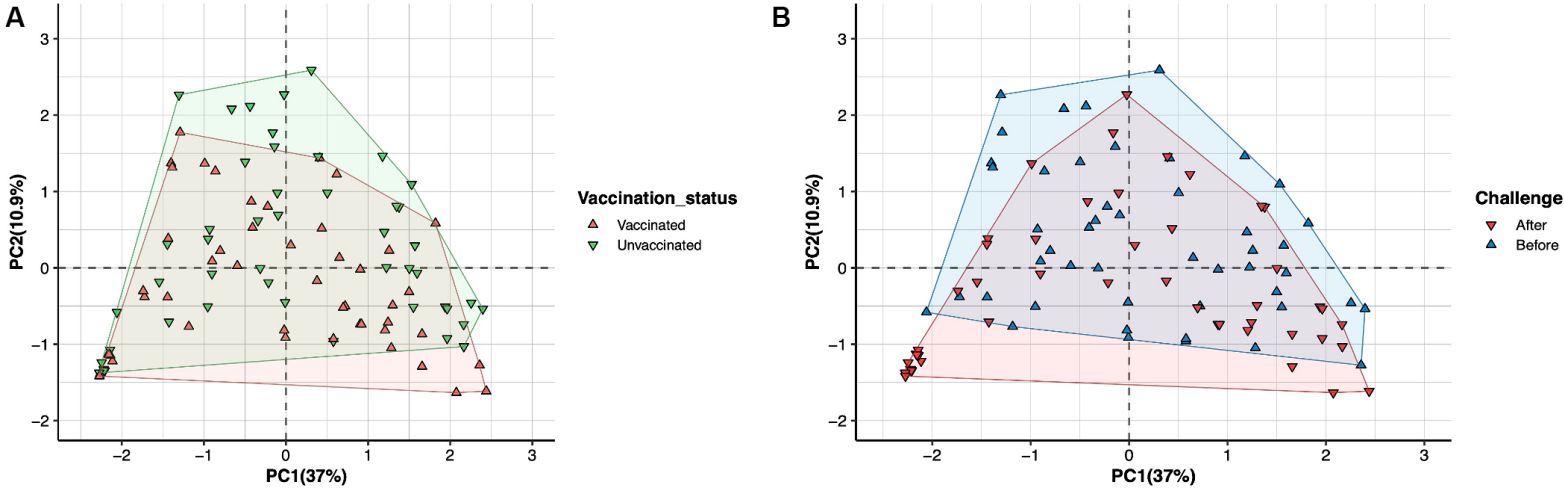
Principal component analysis (PCA) plot describing the variance of the vaginal microbiome of heifers in **(A)** vaccinated (green) and unvaccinated (red) heifers. **(B)** Before challenge (blue) and after challenge with *Campylobacter fetus* subsp. *venerealis* (red). No variation was observed in vaccinated and unvaccinated heifers before and after challenge. The PC1 variance was 37%, and PC2 variance was 10.9%.

No significant changes in microbial diversity associated with the oestrous cycle (oestrus and dioestrus) and challenge (*p* > 0.05) in vaccinated and unvaccinated heifers were observed based on the Shannon diversity index. However, a higher diversity of bacteria was identified in animals before challenge than after challenge, regardless of the cycle stage. In vaccinated heifers, the diversity of the microbiomes decreased after challenge than before challenge during oestrus (1.35 ± 0.52 vs. 3.33 ± 109), while in unvaccinated heifers microbial diversity was slightly higher in dioestrus after challenge (2.68 ± 0.99) than before challenge (1.98 ± 0.84) (Figure 3). Furthermore, PCA did not identify any cluster variations of the microbiome of vaccinated and unvaccinated heifers before and after challenge (Figures 4A,B). The PC1 and PC2 accounted for 37 and 10.9% of the variance in the vaginal microbiome (Permutation test, *p* < 0.001).

#### 3.3.2 Dysbiosis in vaginal microbiome and biomarkers for BGC

**Figure 5:**
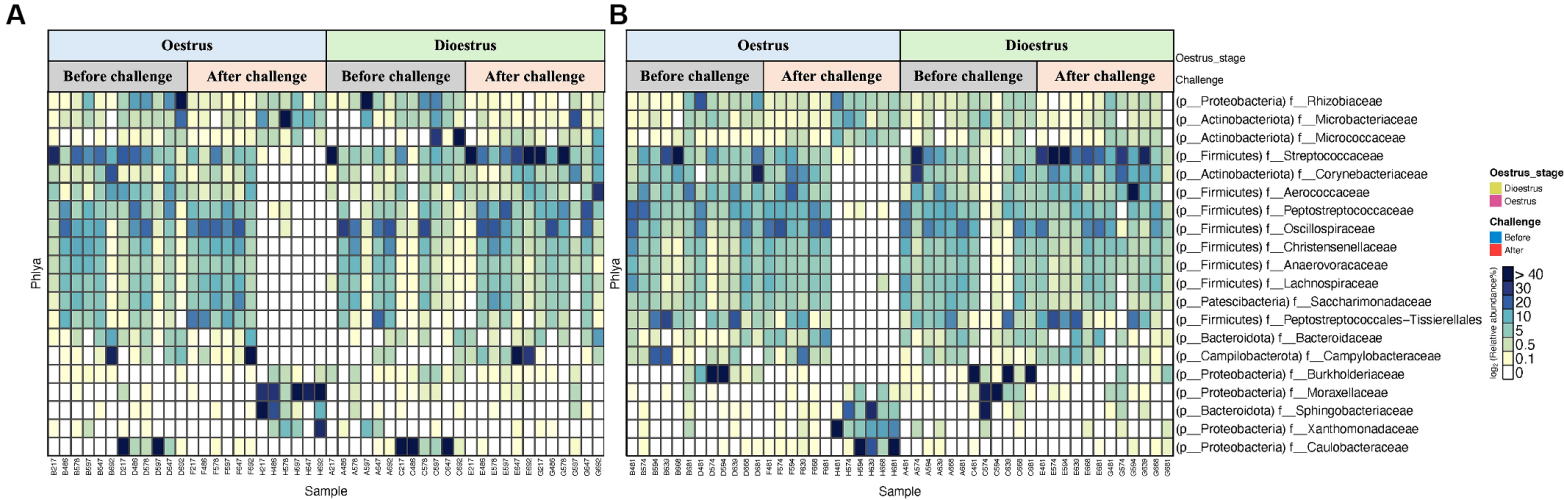
Heat map representing the top 20 most abundant phyla of vaginal 16S rRNA (V5–V8 region amplicons) microbiome associated with oestrous and dioestrous and challenge before and after with *Campylobacter fetus* subsp. *venerealis* in **(A)** unvaccinated and **(B)** vaccinated heifers.

**Figure 6:**
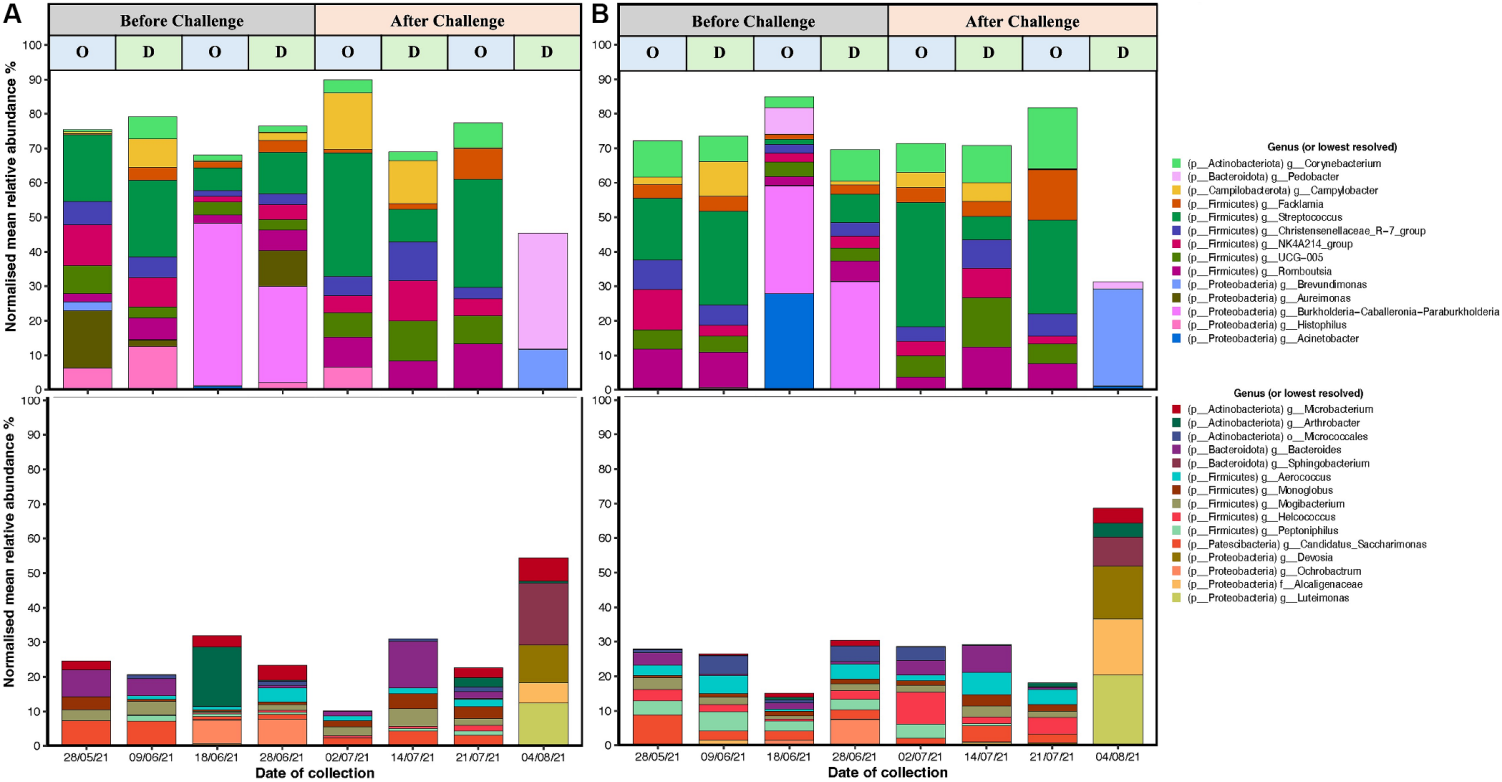
Top 20 high abundant (upper) and low abundant (lower) genus from 16S rRNA (V5–V8 region amplicons) microbiome analyses associated with *C. fetus* subsp. *venerealis* challenge, and oestrous (“O”) vs. dioestrous (“D”) cycle stage in **(A)** unvaccinated and **(B)** vaccinated heifers. *X*-axis is the time of sample collection before challenge (28/05/2021, 09/06/21, 18/06/21, and 28/06/2021) and after challenge (2 days: 02/07/21, 2 weeks: 14/07/21, 3 weeks: 21/07/21, and 4 weeks: 04/08/2021), and *y*-axis is presenting the normalised mean relative abundances of the genus. *Campylobacter* spp. reads are highlighted as yellow and have been represented diagrammatically in Figure 7.

A total of 19 phyla were detected in the vaginal microbiome of unvaccinated and vaccinated heifers, and the most common bacterial phyla were Proteobacteria, Firmicutes, Actinobacteriota, Patescibacteria, and Bacteroidota (Figures 5A,B and Supplementary Table S3.1). In both unvaccinated and vaccinated heifers, Proteobacteria (98%) were higher in abundance before challenge, while after challenge the relative abundances of Firmicutes (90%) and Actinobacteriota (53%) increased. Figures 6A,B show the mean relative abundances of the genera in the vaginal microbiome before and after challenge in unvaccinated and vaccinated heifers. At recovery, the abundance of Firmicutes decreased and Bacteroidota and Proteobacteria increased, but Bacteroidota were higher in unvaccinated heifers and Proteobacteria were higher in vaccinated heifers (Supplementary Table S3.2). Proteobacteria were higher in abundance in oestrus, while Firmicutes were higher in dioestrus in both vaccinated and unvaccinated heifers before challenge. The relative abundances of Firmicutes increased, while Proteobacteria decreased in both groups after challenge.

**Figure 7:**
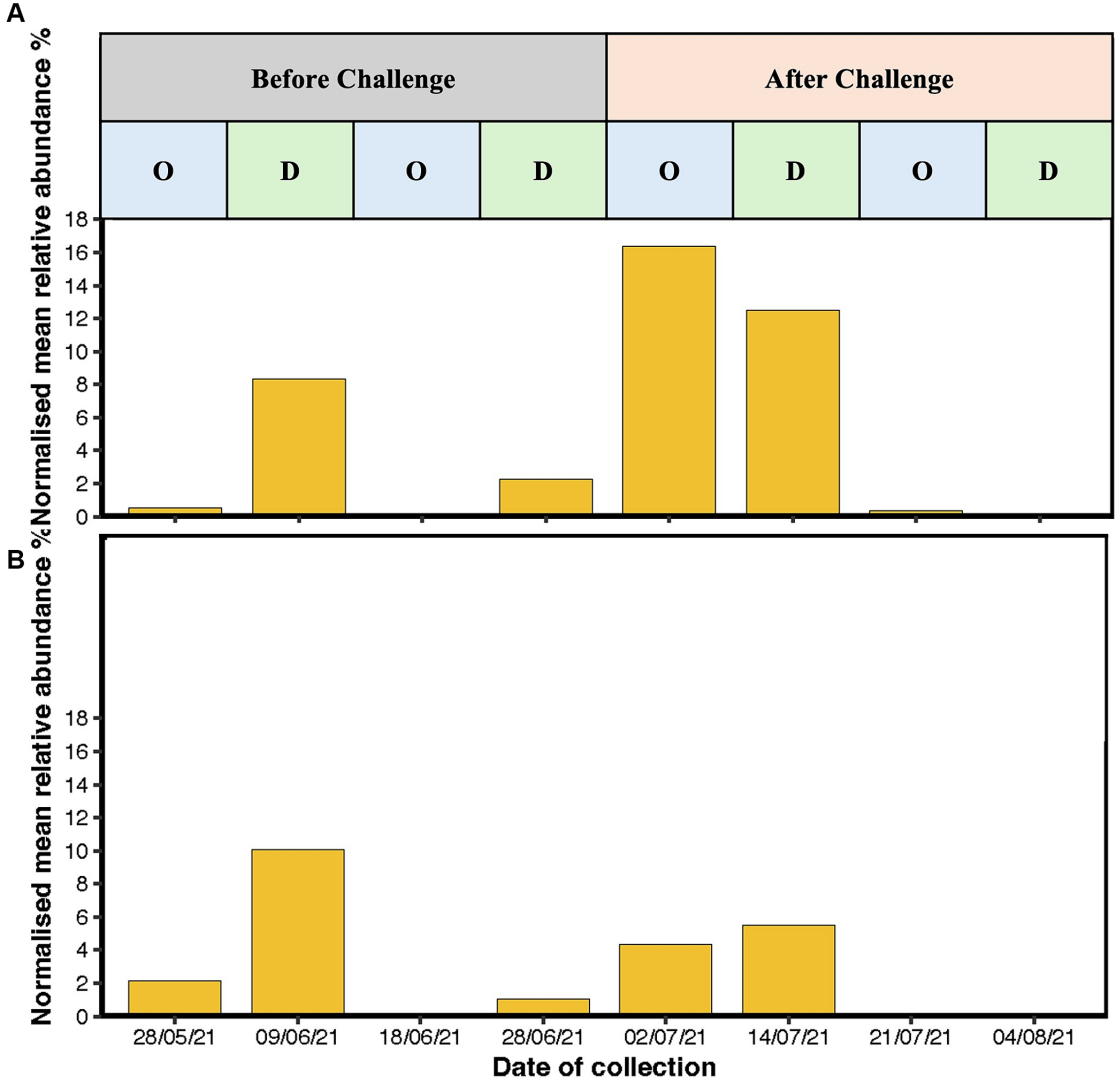
Bar graph representing the mean relative abundance of *Campylobacter* sp. reads in **(A)** unvaccinated heifers and **(B)** vaccinated heifers before and after challenge with *Campylobacter fetus* subsp. *venerealis* (Strain 76,223) with oestrous stages highlighted during the course of the sampling. *X*-axis is presenting the time of sample collection before challenge (28/05/2021, 09/06/21, 18/06/21, and 28/06/2021) and after challenge (2 days: 02/07/21, 2 weeks: 14/07/21, 3 weeks: 21/07/21, and 4 weeks: 04/08/2021), and *y*-axis is presenting the normalised mean relative abundances of the genus.

The most abundant genera were *Streptococcus, Burkholderia-Caballeronia-Paraburkholderia, Arthrobacter*, and *Acinetobacter* (Figures 6A,B). Before challenge, *Burkholderia-Caballeronia-Paraburkholderia* (96%) were the most abundant genera in unvaccinated heifers followed by *Streptococcus* (65%) and *Arthrobacter* (56%), while in vaccinated heifers, *Acinetobacter* (95%) was the most abundant genus followed by *Burkholderia-Caballeronia-Paraburkholderia* and *Streptococcus* spp. *Streptococcus* spp. had a higher abundance in oestrus compared to dioestrus after challenge in both vaccinated and unvaccinated animals. The relative abundances of *Corynebacterium* increased after challenge in vaccinated heifers. The level of Proteobacteria is dependent on the cycle stage with higher abundances during oestrus. In addition, *Arthrobacter* sp. changed during the oestrous cycle consistently with higher abundances in oestrus than dioestrus. *Campylobacter* spp. increased in abundance after challenge in both groups and was higher in the unvaccinated group. *Campylobacter* spp. was highest at 2 days post-challenge in the unvaccinated group while highest in the vaccinated group at 2 weeks after challenge. At 4 weeks post-challenge, *Campylobacter* spp. was absent in both unvaccinated and vaccinated heifers (Figures 7A,B).

### 3.4 Protein identification and immune biomarkers for BGC

**Figure 8:**
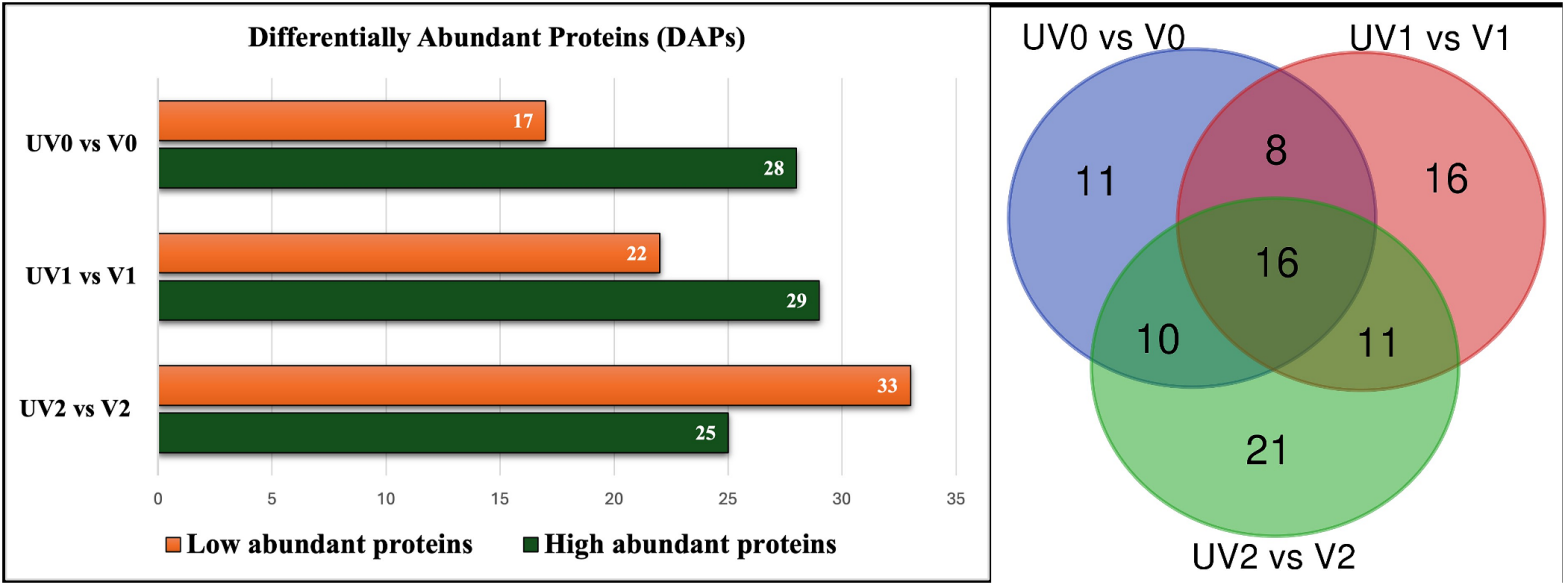
Bar graph illustrating the total number of high abundant and low abundant differentially abundant proteins (DAPs) with log2FC < 0.2 and adjusted *p* < 0.05 and Venn diagram describing the number of commonly found DAPs in vaccinated and unvaccinated heifers in three time points—post-vaccination (UV0 vs. V0), post-challenge (UV1 vs. V1), and post-recovery (UV2 vs. V2).

**Figure 9:**
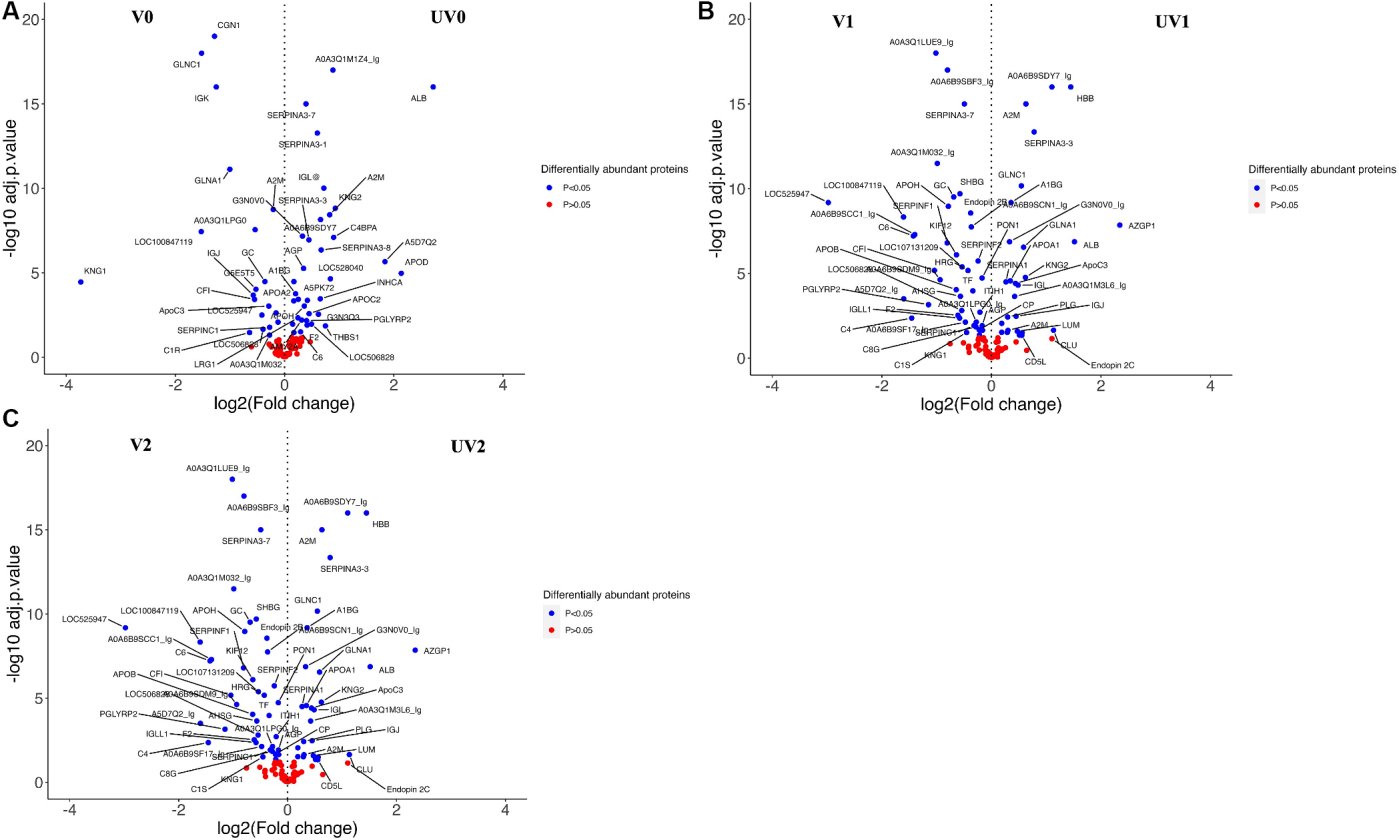
Volcano plots representing the changes in differentially abundant proteins (DAPs, log2FC > 0.2; adjusted *p* < 0.05) associated with vaccine challenge at three different time points—**(A)** pre-challenge (UV0 vs. V0), **(B)** post-challenge with *Campylobacter fetus* subsp. *venerealis* (UV1 vs. V1), and **(C)** post-recovery (UV2 vs. V2). +(ve) values are highly abundant proteins in the unvaccinated group (UV), and −(ve) values are highly abundant proteins in the vaccinated group (V). Blue-dotted proteins are significantly different in abundance (adjusted *p* < 0.05), and red dots are non-significant proteins (adjusted *p* > 0.05).

A total of 210 unique serum proteins were identified by ProteinPilot software (SCIEX^®^ 5.02) (Supplementary Table S4.1). The relative abundance of each protein within each group was quantified with an FDR cutoff value of 1%, and a total of 130 unique proteins was identified after Peakview 2.1 (SCIEX^®^) analysis (Supplementary Tables S5.1–S5.7). The serum proteomes of unvaccinated and vaccinated heifers were compared at three time points, and significant proteins (adjusted *p* < 0.05) with log2FC more than 0.2 were considered as significantly differentially abundant proteins (DAPs) in each group for further analysis. The total numbers of DAPs found in pre-challenge (UV0 vs. V0), post-challenge (UV1 vs. V1), and post-recovery (UV2 vs. V2) groups were 45, 51, and 58, respectively, in which 28, 29, and 25 proteins were highly abundant in unvaccinated heifers (Figures 8, 9A–C and Supplementary Tables S6.1–S6.3).

Depending on the log2FC values, the most abundant proteins found in unvaccinated heifers before challenge were albumin (B0JYQ0; log2FC = 2.7), apolipoprotein D (APOD; log2FC = 2.1), and immunoglobulin (Ig) alpha 2 heavy chain (A5D7Q2_IgM; log2FC: 1.8). Kininogen-II (P01045; KNG2) and two complement components (A5D9D2; C4BPA and EIB805; C3) were also significantly higher in abundance (log2FC = 0.9, 0.9, 0.8, respectively) in the pre-challenge unvaccinated group. While kininogen-1 (KNG1; log2FC = 3.7), Ig-like domain-containing protein (F1MLW8; log2FC = 1.5), globin C1 (GLNC1; log2FC = 1.5), conglutinin (CGN1; log2FC = 1.3), and vitamin D-binding GC protein (Q3MHN5; log2FC = 0.4) were significantly higher in vaccinated pre-challenge heifers.

In the post-challenge comparison, albumin (ALB; log2FC = 1.4), KNG2 (log2FC = 1.4), and complement components, namely, C8G, C4BPA, C6, and C3, were highly abundant proteins in unvaccinated heifers. Haptoglobin homologs (HP-20 and HP-25) also had a significant abundance in post-challenge unvaccinated heifers. In the vaccinated post-challenge, serotransferrin (Q29443; log2FC = 1.8) was identified as the top high abundant significant protein along with immunoglobulin lambda-1 light chain (F1MLW8; log2FC = 1.4), endopin 2C (Q32T06; log2FC = 1.0), and apolipoprotein D (F1MS32; log2FC = 0.9). The abundance of IgG was higher in the unvaccinated heifers after challenge and increased in the vaccinated heifers after recovery. Globin C1, globinA1, and conglutinin were highly abundant proteins in pre-challenge and post-challenge vaccinated heifers while identified as highly abundant proteins in post-recovery unvaccinated heifers. Unvaccinated heifers also showed higher abundances of zinc alpha 2 glycoprotein (AZGPI; log2FC = 2.3), clusterin (CLU; log2FC = 1.1), alpha 2-macroglobulin (A2M; log2FC = 0.6), and CD5 antigen-like (CD5L; log2FC = 0.6) proteins after recovery.

**Figure 10:**
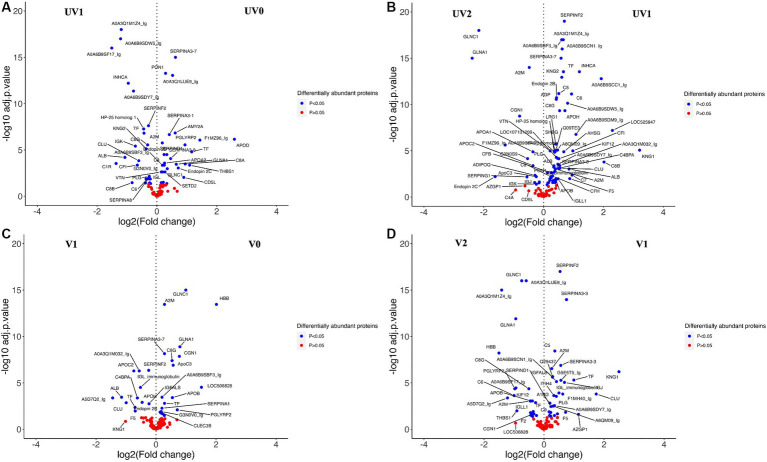
Volcano plots comparing DAPs within groups between **(A)** between unvaccinated heifers before and after challenge (UV0 vs UV1), **(B)** between unvaccinated heifers after challenge and after recovery (UV1 vs UV2), **(C)** between vaccinated heifers before and after challenge (V0 vs V1), and **(D)** between vaccinated heifers after challenge and after recovery (V1 vs V2). Blue dotted proteins are significantly different in abundance (adjusted *p* < 0.05) and red dots are non-significant proteins (adjusted *p* > 0.05).

When comparing the relative abundance of proteins within groups before and after challenge, the number of DAPs was lower in vaccinated (V0 vs. V1; *n* = 25) than the unvaccinated (UV0 vs. UV1; *n* = 43) (Figures 10A,C and Supplementary Tables S6.4, S6.5). Within UV0 vs. UV1 comparison, kininogen-1 (log2FC = 4.7), fetuin B (FETUB; log2FC = 1.4), C8B (log2FC = 0.8), HP homolog 25 (log2FC = 0.4), and uncharacterised proteins “identified as IgG after BLAST search” (A0A6B9SF17, A0A6B9SDW5, A0A3Q1M1Z4; log2FC = 15, 1.2, 1.2) increased in response to challenge. In the vaccinated group, IgG (A5D7Q2; log2FC = 1.4), albumin (log2FC = 1.1), clusterin (CLU; log2FC = 1.0), apolipoprotein C II (log2FC = 0.7), serotransferrin (log2FC = 0.7), and coagulation factor-5 (F5; log2FC = 0.7) increased in response to challenge. Comparing post-challenge with post-recovery, a total of 68 and 49 differentially abundant proteins were identified in the unvaccinated (UV1 vs. UV2) and vaccinated (V1 vs. V2) groups, respectively (Figures 10B,D; Supplementary Tables S6.6, S6.7). In unvaccinated heifers (UV1 vs. UV2), the abundances of complement components (C8B, CF1, C7, C6, C8G, C5, and C4BPA; log2FC = 2.0, 1, 1, 0.9, 0.5, 0.5, 0.5) and coagulation factor (F5; log2FC = 0.9) significantly decreased in post-recovery heifers. In vaccinated heifers, the abundances of KNG1, CLU, C5, and F5 were also reduced in post-recovery heifers, while C6, C8G, and F2 increased in abundance.

#### 3.4.1 Enrichment analysis of identified serum proteins

**Figure 11:**
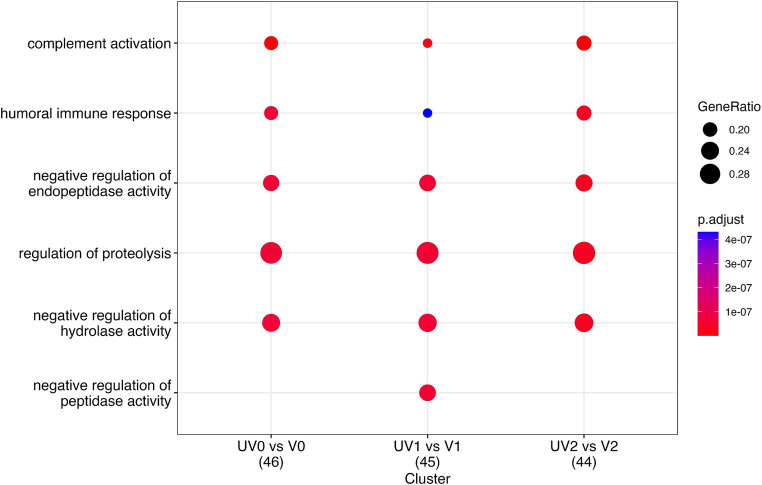
Dot plot describing the most important pathways of identified proteins through gene enrichment analysis of DAPs in vaccinated (V) and unvaccinated (UV) heifers at three different time points—post-vaccination (UV0 vs. V0), post-challenge (UV1 vs. V1), and post-recovery (UV2 vs. V2). The size of the dots denotes the number of genes involved, and the colour code indicates the level of significance using Benjamini–Hochberg corrections.

The comparison of serum proteins in vaccinated and unvaccinated heifers at three different time points (pre-, post-challenge, and post-recovery) identified the enrichment of proteins associated with several important biological processes. These include complement activation (GO:0006956; GO:0006958; GO:0006959), humoral immune responses (GO:0006959), blood coagulation (GO:0007596; GO:0050817), inflammatory responses (GO:0006954), adaptive immune responses (GO:0002250), defence response to other organism (G:0098542), negative regulation of endopeptidase (GO:0010951) and peptidase (GO:0010466) activity, and regulation of proteolysis (GO:0030162) (Figure 11; Supplementary Tables S7.1–S7.3). In addition, complement activation and the coagulation cascade activation pathways were identified in the KEGG pathway analyses.

When comparing pre-challenge vaccinated with unvaccinated groups of heifers, F2, THBS1, APOH, APOA2, C6, C4BPA, thrombospondin 1 (THBSI), C3, and IgG domain-containing proteins (G3NOVO, G3N3Q3) were significantly high in abundance in unvaccinated and KNG1, CF1, G3E513, and JCHAIN proteins in the vaccinated group. These proteins are associated with blood coagulation, complement activation, defence responses, adaptive immune responses, and inflammatory responses. TF, APOD, ADIPOQ, G5E513, JCHIAN, G3N3Q3, and G3NOVO highly abundant significant proteins in vaccinated heifers and ALB, C3, C6, and C8G in unvaccinated heifers were associated with complement activation, defence mechanisms, and humoral immune responses in post-challenge comparison.

**Figure 12:**
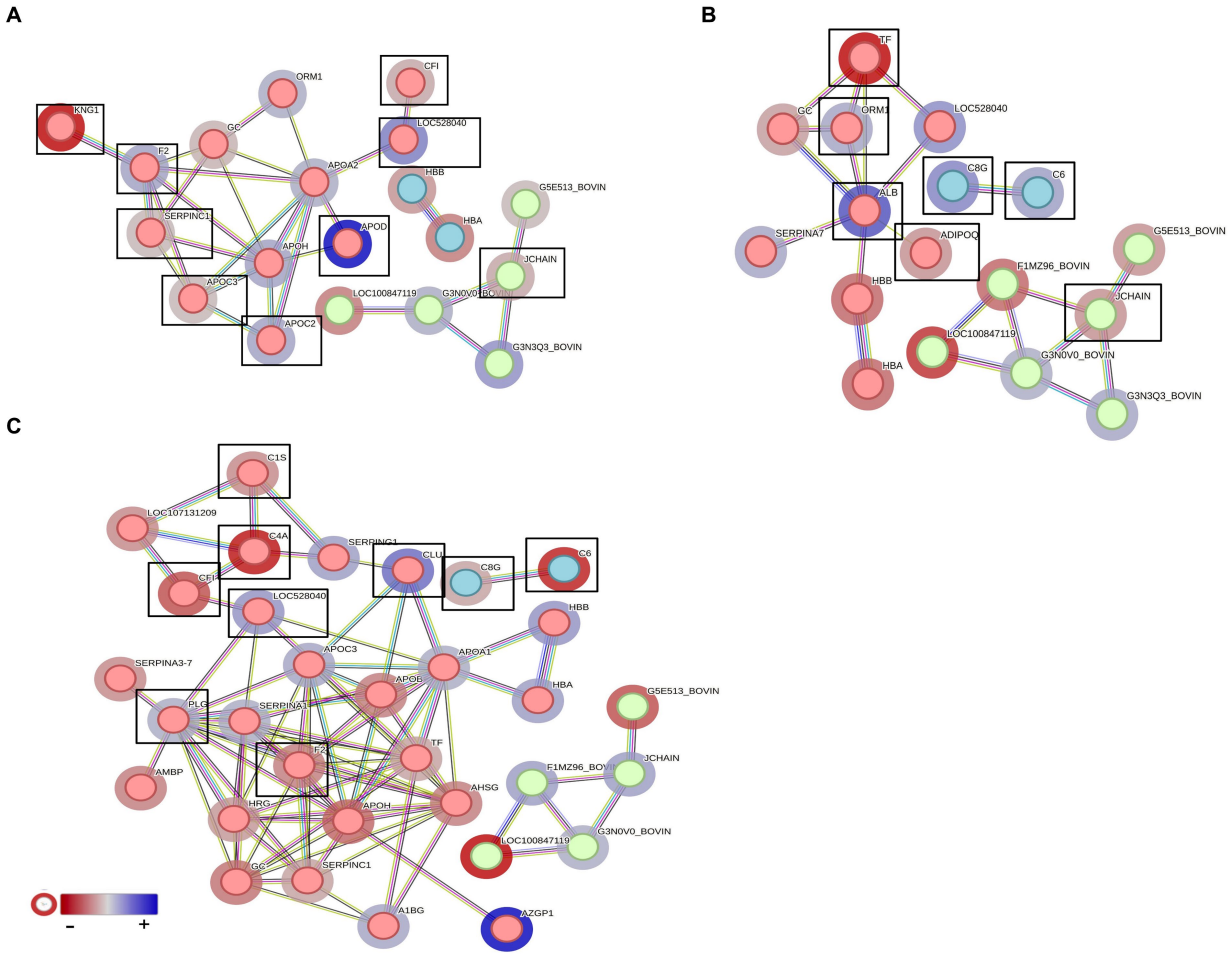
STRING analysis presenting the protein–protein interactions in the vaccinated and unvaccinated groups at **(A)** pre-challenge (UV0 vs. V0), **(B)** post-challenge (UV1 vs. V1), and **(C)** post-recovery (UV2 vs. V2). Each node represents an individual protein. *k*-mean clusters showing strong interactions are highlighted as “red,” “green,” and “cyan blue” coloured nodes. The halo colour is based on the log2FC value of proteins. Important proteins associated with complement activation and blood coagulation are indicated with box.

In the post-recovery stage, vaccinated cattle exhibited an increase in proteins associated with complement activation, with notable proteins being C6, C4A, C8G, and CF1, while unvaccinated cattle showed higher levels of proteins related to blood coagulation, such as PLG, CLU, and SERPING1. Clusterin (CLU) is a significantly higher DAP found in vaccinated and unvaccinated post-challenge and post-recovery heifers and is associated with proteolysis (GO:0006508), immune effector process (GO: 0002252), and positive regulation of response to stimulus (GO:0048584) (Figures 12A–C). Vaccination appeared to enhance various immune responses and defence mechanisms in heifers, particularly evident in the post-challenge and post-recovery stages.

## 4 Discussion

Key advancements in microbiome and proteomics have shed light on the pathogenesis of complex cattle diseases, particularly reproductive inflammatory diseases. Bovine genital campylobacteriosis (BGC), caused by *C. fetus venerealis*, is a significant reproductive disease worldwide, resulting in production and pregnancy losses. Despite its impact, the vaginal microbiome dysbiosis in heifers due to this infection and immune biomarkers for BGC have not been previously characterised. This study utilised 16S rRNA amplicon sequencing and quantitative proteomics to explore the vaginal microbiome and serum proteins of heifers in response to *C. fetus venerealis* infection to identify host biomarkers for BGC.

### 4.1 Characterisation of the microbiome of vaccinated and unvaccinated heifers for BGC

The bovine vaginal microbiome is known to be dominated by bacteria from the Firmicutes, Bacteroidota, and Proteobacteria phyla (Laguardia-Nascimento et al., 2015). The abundances of these bacterial phyla are associated with different factors including oestrous cycle, pregnancy, days of post-partum and post-partum uterine diseases, breed, age, and environment (Wang et al., 2018b; Ault et al., 2019a,b; Quereda et al., 2020; Gohil et al., 2022). The bovine reproductive tract microbiome is a reflection of environmental organisms presented in soil, skin, and water-confirmed in metagenomics studies published in Scientific Reports in 2022 (Ong et al., 2022a). In our study, vaccination against *C. fetus venerealis* did not affect the phyla of the vaginal microbiome of heifers with both vaccinated and unvaccinated heifers dominated by Proteobacteria, Firmicutes, Actinobacteriota, and Bacteroidota before challenge. Proteobacteria and Firmicutes were identified as the highest abundant bacteria in healthy heifers which is similar to studies reported by Tasara et al. (2023). Giannattasio-Ferraz et al. (2019) found Firmicutes as the dominant bacterial phylum followed by Bacteroidota, Proteobacteria, and Actinobacteria when characterising the vaginal microbiomes of healthy Gyr and Nellore cattle breeds.

Here, we have shown that the microbiome changes after a live challenge, with *C. fetus venerealis*. The mean abundance of Proteobacteria decreased, while Firmicutes and Campylobacterota increased. An increased abundance of Firmicutes is known to help to prevent the growth of pathogens by reducing the pH of vaginal mucus (Vieco-Saiz et al., 2019). Interestingly, we did not find an increase in Bacteroidota, Fusobacteria, and Porphyromonas in vaccinated heifers after challenge even though these phyla have been synergistically associated with post-partum clinical infections such as metritis, endometritis, and cervicitis (Wang et al., 2018a; Galvão et al., 2019, reviewed by Ong et al., 2021).

Firmicutes, especially *Streptococcus* spp., increased during oestrus over dioestrus after challenge in both vaccinated and unvaccinated heifers. *Streptococcus* spp. was more abundant in vaccinated than unvaccinated individuals. The presence of *Streptococcus* spp. has been shown to promote uterine health and is negatively correlated with uterine inflammation and pathogenic bacteria such as *Trueperella pyogenes* (Williams et al., 2005; Gilbert and Santos, 2016). From previous studies, it was observed that the bovine vaginal microbiome is hormone-dependent, and the heterogenicity in microbial communities observed during luteal and follicular phases impacts the vaginal microbiome of heifers (Laguardia-Nascimento et al., 2015; Quereda et al., 2020; Gohil et al., 2022). It has been observed that microbial phyla such as Firmicutes, Actinobacteria, and Bacteroidota are found to be prominent during oestrus in some cattle/buffalo, with a reduction in the Proteobacteria, which is high during dioestrus (Mahalingam et al., 2019). An increased abundance of *Corynebacterium* (Actinobacteriota) was found in the vaccinated heifers after challenge compared to unvaccinated in our study. *Corynebacterium* spp. (non-diphtheria) can produce bacteriocin, an antimicrobial agent which regulates vaginal microbiocenosis in humans and may prevent the growth of opportunistic bacteria (Gladysheva et al., 2022).

In this study, the challenge with *C. fetus venerealis* resulted in a shift in the heifer vaginal microbiomes, no clinical disease, and a return to oestrus. The mean abundance of *Campylobacter* spp. increased after challenge in both vaccinated and unvaccinated heifers. Although the abundance of *Campylobacter* was higher in unvaccinated heifers than vaccinated, the vaccinated heifers cleared the infection earlier as confirmed using qPCR. A recent study on the cervicovaginal microbiota of female beef cattle harbouring *C. fetus venerealis* showed that positive and negative heifers had similar bacterial composition and that the presence of *C. fetus venerealis* did not affect cyclicity which is similar to our study (De Carli et al., 2023). Normally, female cows infected with *C. fetus venerealis* can clear the infection and gain mucosal immunity within 3–5 months of infection. However, immunity is short-lived, and cows with compromised immunity often fail to return to oestrus (Bondurant, 2005).

Although 16S amplicon sequencing can provide an insight into microbial dysbiosis, it has some limitations such as the inability to identify to the species level and the biased amplification of 16S rRNA due to the variability of 16S genomic copy numbers in different species. Metagenomics by adaptive sampling is a long-read sequencing method that depletes host DNA to provide more accurate and unbiased profiling (Ong et al., 2022a,b). A further limitation of this study is the small number of animals used for the trial. It would be of interest to validate the results in a larger field study, utilising metagenomic adaptive sampling to further determine the association of BGC immunity with the vaginal metagenome.

### 4.2 Heifer immune biomarkers for BGC

To identify vaccination biomarkers, we compared the serum proteomic profile to identify differentially abundant proteins between the unvaccinated and vaccinated heifers before challenge. Conglutinin was highly abundant in the vaccinated group post-challenge. Conglutinin is an important immune response protein particularly associated with innate immunity. It plays a crucial role in recognising and eliminating pathogens by binding to the sugar surface of microorganisms and facilitates their removal by iC3b opsonin, a degradation product of the complement system, which is deposited on microbial cell surfaces (Friis-Christiansen et al., 1990; Dec et al., 2012). The comparison of serum proteomes of unvaccinated heifers following challenge (UV0 vs. UV1) identified several interesting biomarker candidates including kininogen I, fetuin B, complement components (C6, C3, C8B, C8G, and CF1), and HP-25 homologs in response to *C. fetus venerealis* infection. Fetuin B (a cystatin) inhibits ovastacin, which has been shown to be involved in zona pellucida hardening, thus interfering with fertilisation (Dietzel et al., 2013). Furthermore, fetuin B inhibits Meprin, which has been shown to modulate the immune system by processing and activating pro-inflammatory cytokines and chemokines that in turn induce the migration of leukocytes to sites of injury or infection (Herzog et al., 2019).

To characterise the immune response to *C. fetus venerealis* infection, we compared the serum proteomes of post-vaccination with those post-challenge from unvaccinated and vaccinated heifers (UV0 vs. V0 and UV1 vs. V1). Vitamin D-binding protein (DBP) was in high abundance in vaccinated cattle before challenge (UV0 vs. V0) and in unvaccinated cattle after challenge (UV1 vs. V1). Vitamin D-binding protein (DBP) can bind to proteoglycans present in the membrane of immune cells and enhance complement C5a-stimulated chemotactic activity of activated neutrophils (Metcalf et al., 1991). This protein has previously been identified as a biomarker for specific inflammatory diseases, such as cirrhosis (Lisowska-Myjak et al., 2020). Furthermore, DBP acts as an anti-tuberculosis agent, leading to increased bacterial killing by the induction of a group of antimicrobial peptides (Cathelicidins) through the triggering of Toll-like receptors (Liu et al., 2006) and thus has been identified as a potential biomarker for tuberculosis and paratuberculosis in human (Seth et al., 2009).

Protein HP-20 and HP-25 homologs were also observed as important highly abundant proteins when comparing UV1 and V1. The log fold change of these HP homologs increased in unvaccinated heifers after challenge. HP homolog proteins are acute-phase response proteins and are actively associated with phagocytosis and immune responses by inhibiting iron uptake of microbes. These proteins have been identified as important protein biomarkers for both clinical and subclinical endometritis and metritis in cattle (Miller et al., 2019; Menta et al., 2023). Clusterin is another important protein observed in post-challenge unvaccinated and vaccinated heifers in response to *C. fetus venerealis* infection. Log fold change of clusterin was higher in vaccinated (1.7) than unvaccinated (0.5) heifers. Clusterin is a glycoprotein which has diverse functions including stimulation of inflammatory cytokines, complement inhibition, immunity modulation, and cell invasion (Wilson and Zoubeidi, 2017). Research suggests that there is an association between CLU and pregnancy-related conditions such as intrauterine growth restriction and recurrent pregnancy loss in humans (Mohanty et al., 2020). The identified proteins appear to be part of a complex immune response to *C. fetus venerealis* infection. Their differential abundance could serve as potential biomarkers to monitor the infection or immune status of the host.

## 5 Conclusion

The characterisation of heifer vaginal microbiomes through the analysis of 16S rRNA amplicons provided an overview of the shift of the reproductive tract microbiome in response to *C. fetus venerealis* infection. In this study, the vaginal microbiome of vaccinated and unvaccinated heifers was dominated by the same bacterial phyla including Proteobacteria, Firmicutes, Actinobacteria, and Bacteroidota. An increased abundance of Firmicutes and the Campylobacterota genus was observed after challenge and were associated with *C. fetus venerealis* challenge in both vaccinated and unvaccinated heifers. *Streptococcus* spp., *Acinetobacter*, and *Corynebacterium* spp. were altered before and after challenge. These bacteria may be involved in the development of vaginal immunity in response to *C. fetus venerealis* infection in heifers. Conglutinin, clusterin, HP homologs, vitamin D-binding protein, and fetuin B were identified as potential biomarkers for *C. fetus venerealis* infection in heifers and could be used as diagnostic and prognostic biomarkers for BGC immunity in cattle. However, further research is needed to understand the specific roles of these proteins in the context of BGC and their utility as diagnostic or prognostic markers.

## Data availability statement

The microbiome datasets generated during the current study are available in the NCBI sequence read archive (SRA) database under BioProject PRJNA1119727 and BioSamples SRR29272614 to SRR29272709. The mass spectrometry proteomics data have been deposited to the ProteomeXchange Consortium via the PRIDE (Perez-Riverol et al., 2022) partner repository with the dataset identifier PXD052863. Data will be accessible using project accession no.: PXD052863 with Token: DEBWJSDFKwZP.

## Ethics statement

The animal study was approved by the University of Queensland Animal Ethics Committee. The study was conducted in accordance with the local legislation and institutional requirements.

## Author contributions

MJ: Data curation, Formal analysis, Investigation, Writing – original draft. AR: Formal analysis, Methodology, Supervision, Writing – review & editing. MF: Formal analysis, Methodology, Supervision, Writing – review & editing. HS: Methodology, Supervision, Writing – review & editing. GF: Conceptualization, Investigation, Supervision, Writing – review & editing. JM: Investigation, Writing – review & editing. GB-H: Conceptualization, Funding acquisition, Investigation, Methodology, Supervision, Writing – review & editing. AT: Conceptualization, Funding acquisition, Supervision, Writing – review & editing.
