# Supplementary material for: Characterisation of reproductive tract microbiome and immune biomarkers for bovine genital campylobacteriosis in vaccinated and unvaccinated heifers
Source: Front Microbiol. 2024 Aug 19;15:1404525. doi: 10.3389/fmicb.2024.1404525 (PMC11366586; doi:10.3389/fmicb.2024.1404525)
Supplement: Supplementary file 1 [file Data_Sheet_1.docx]

| 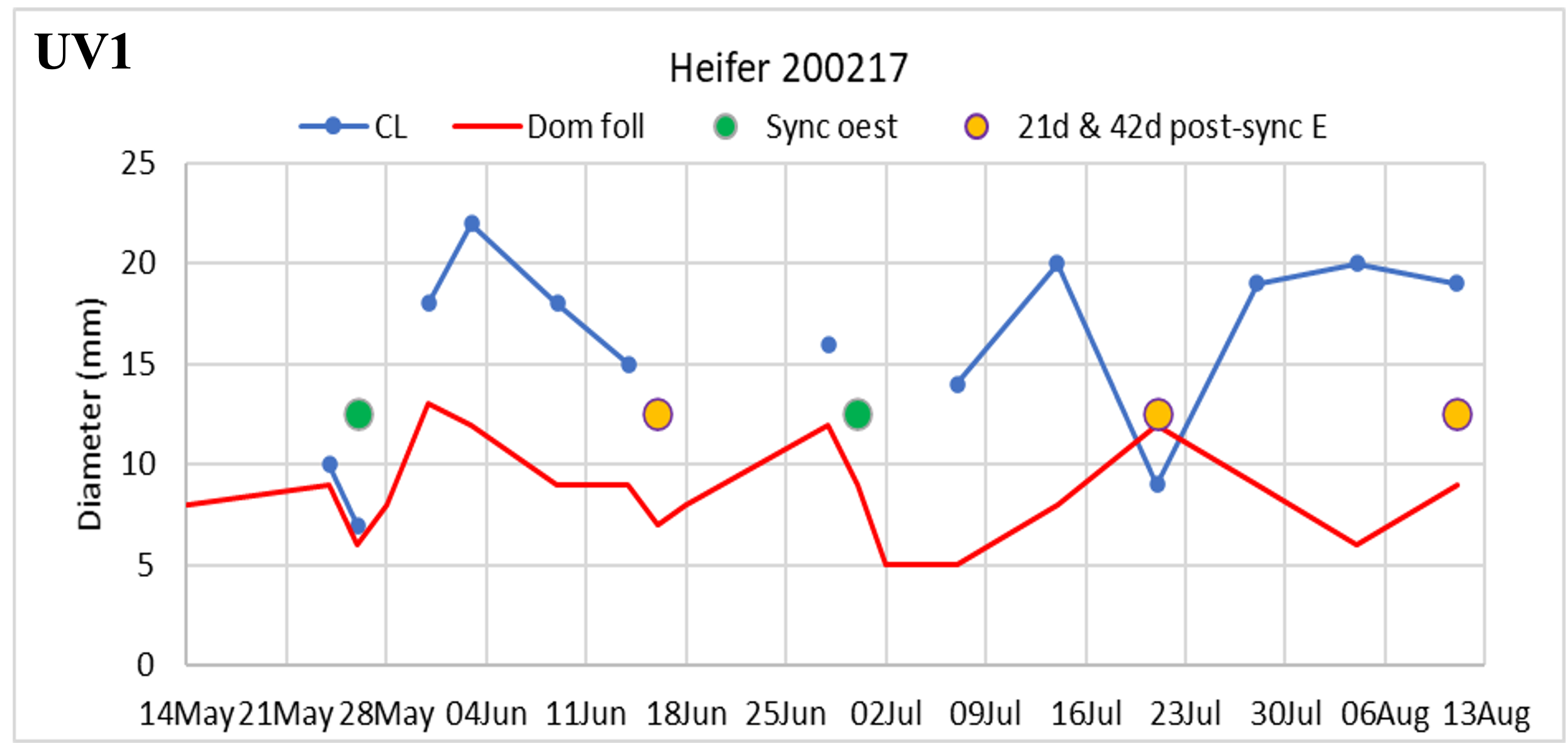 | 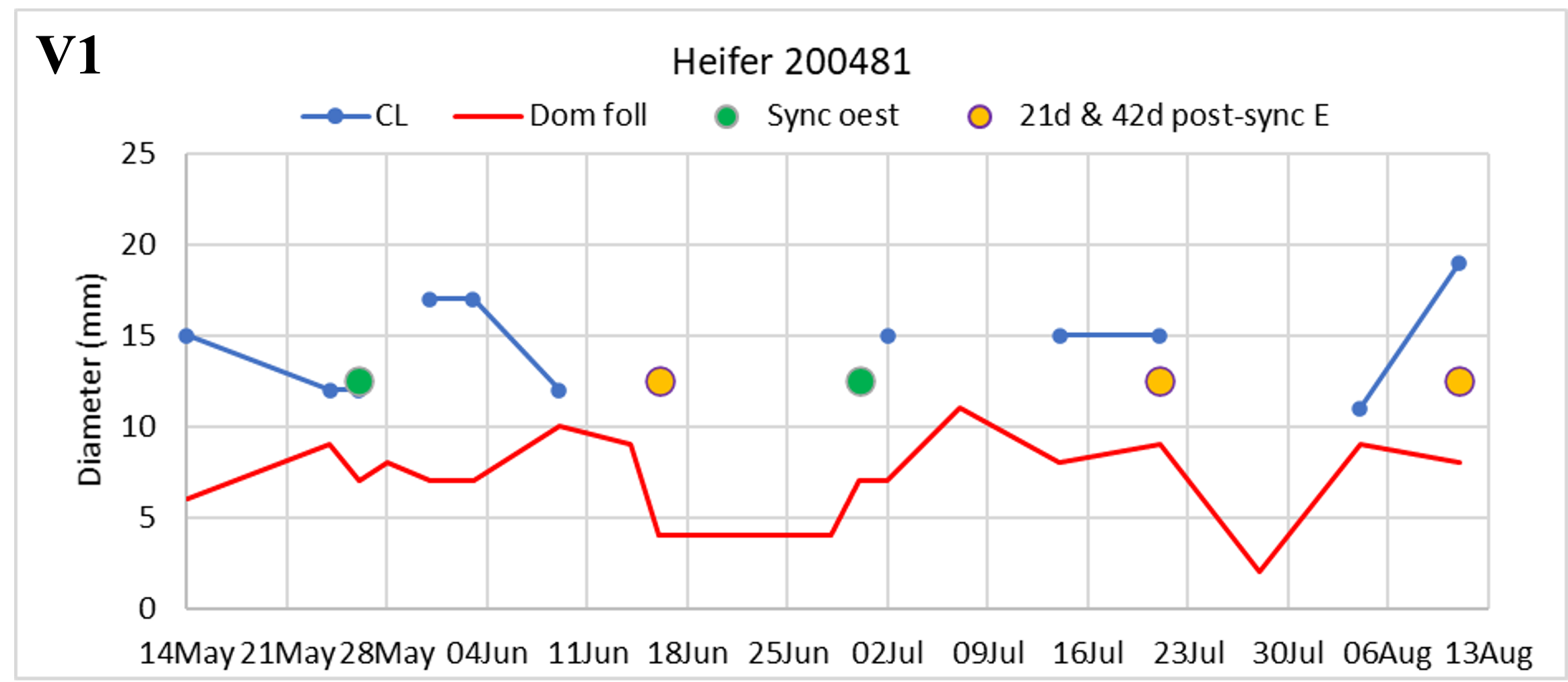 |
| --- | --- |
| 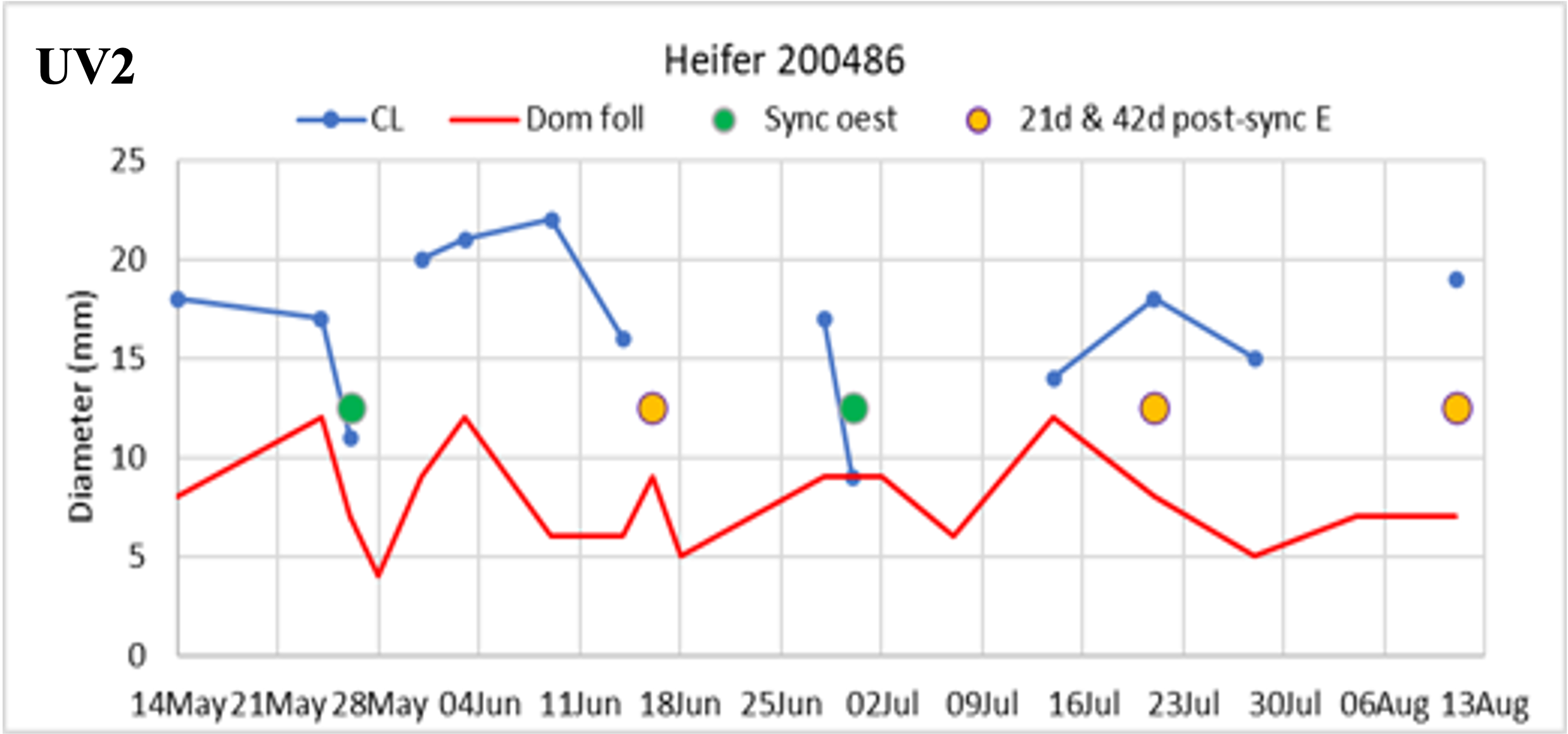 | 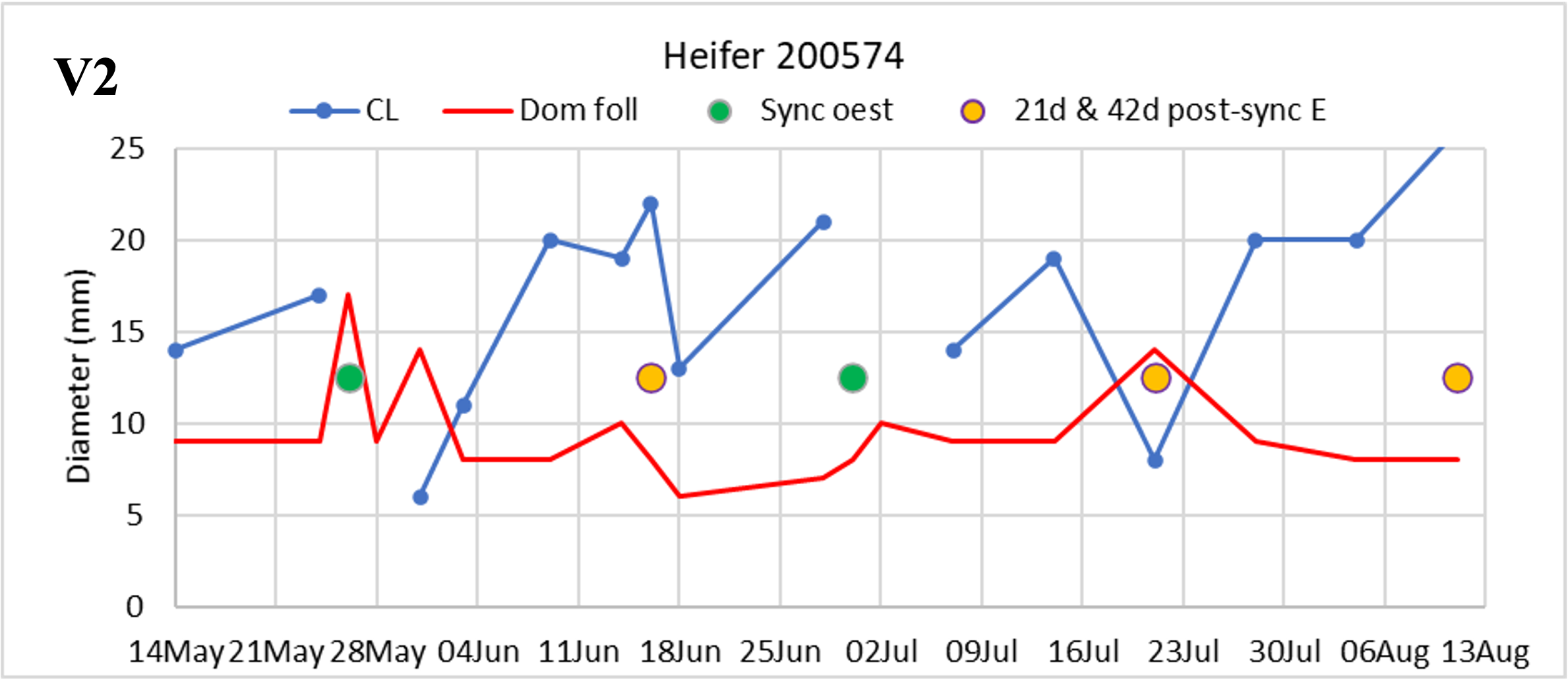 |
| 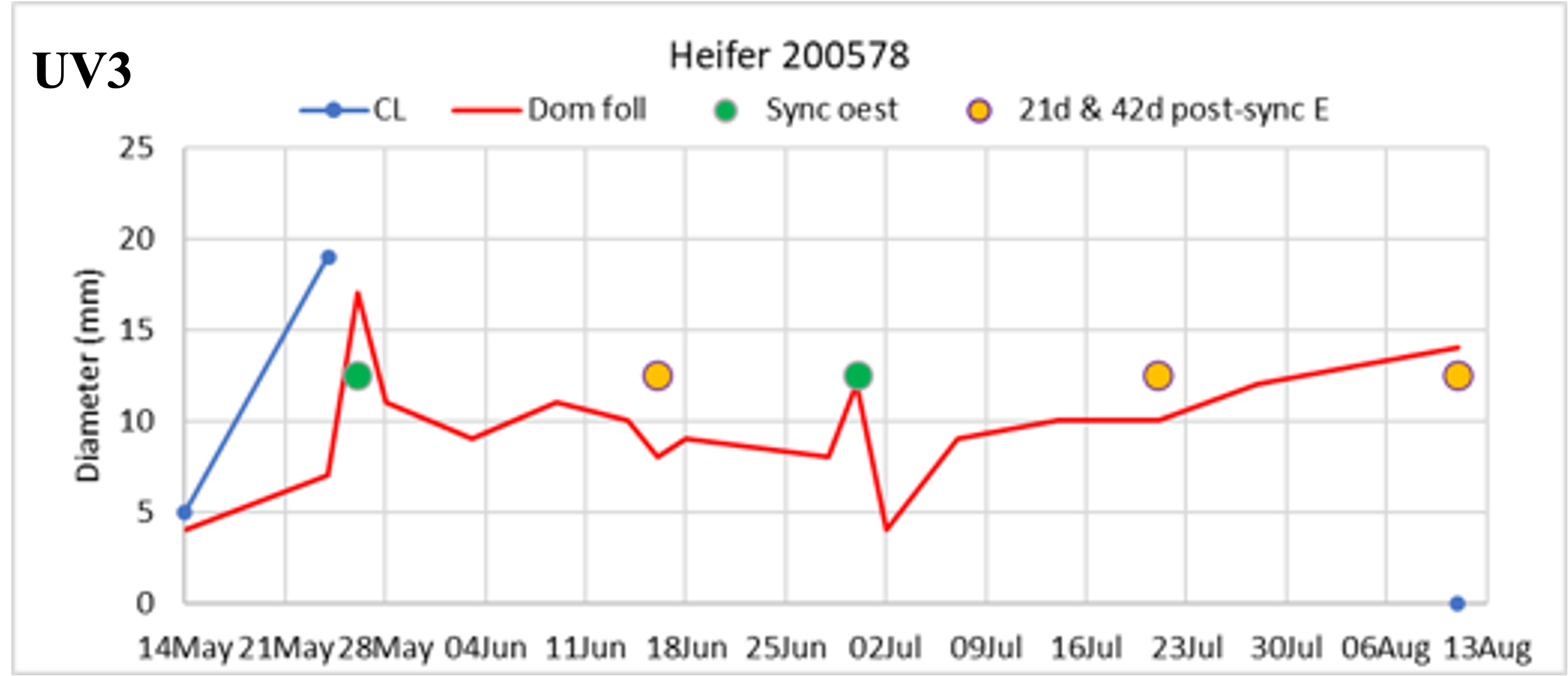 | 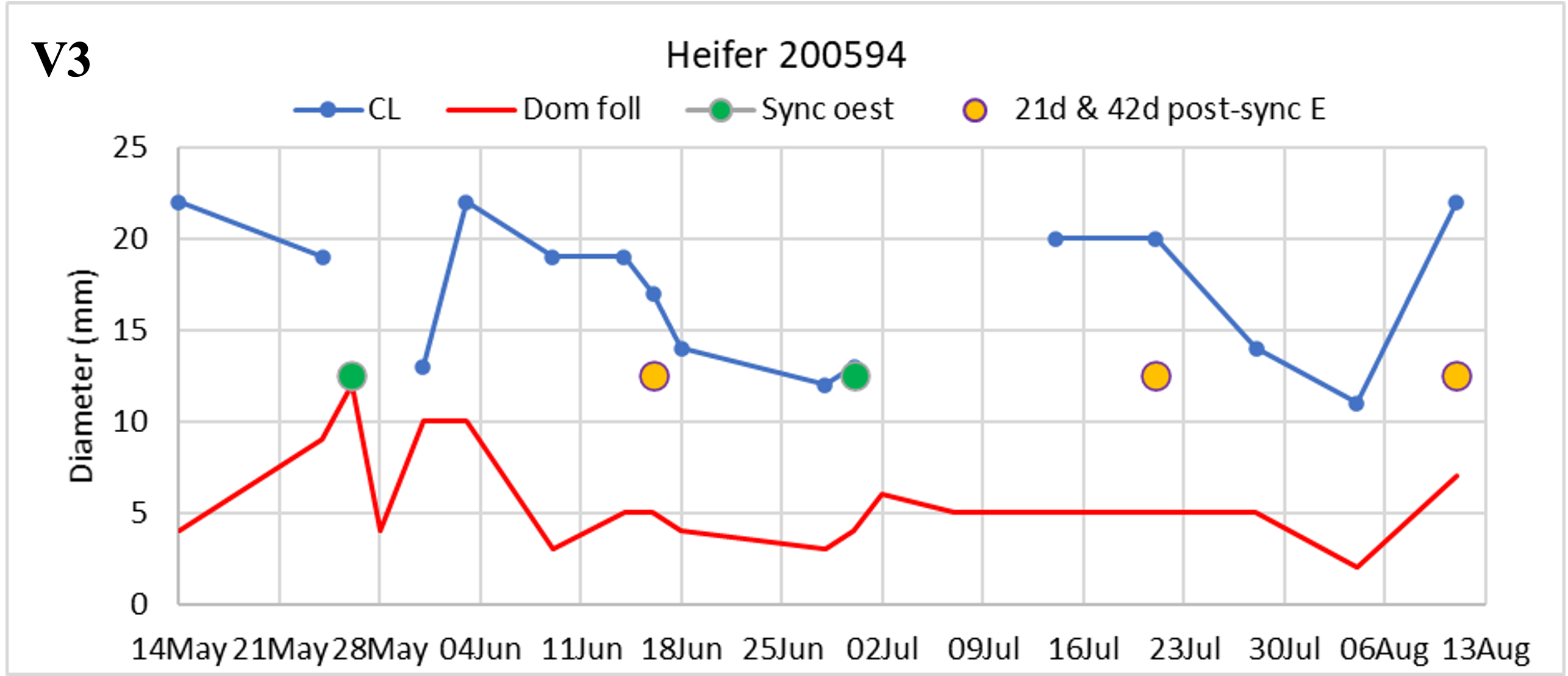 |
| 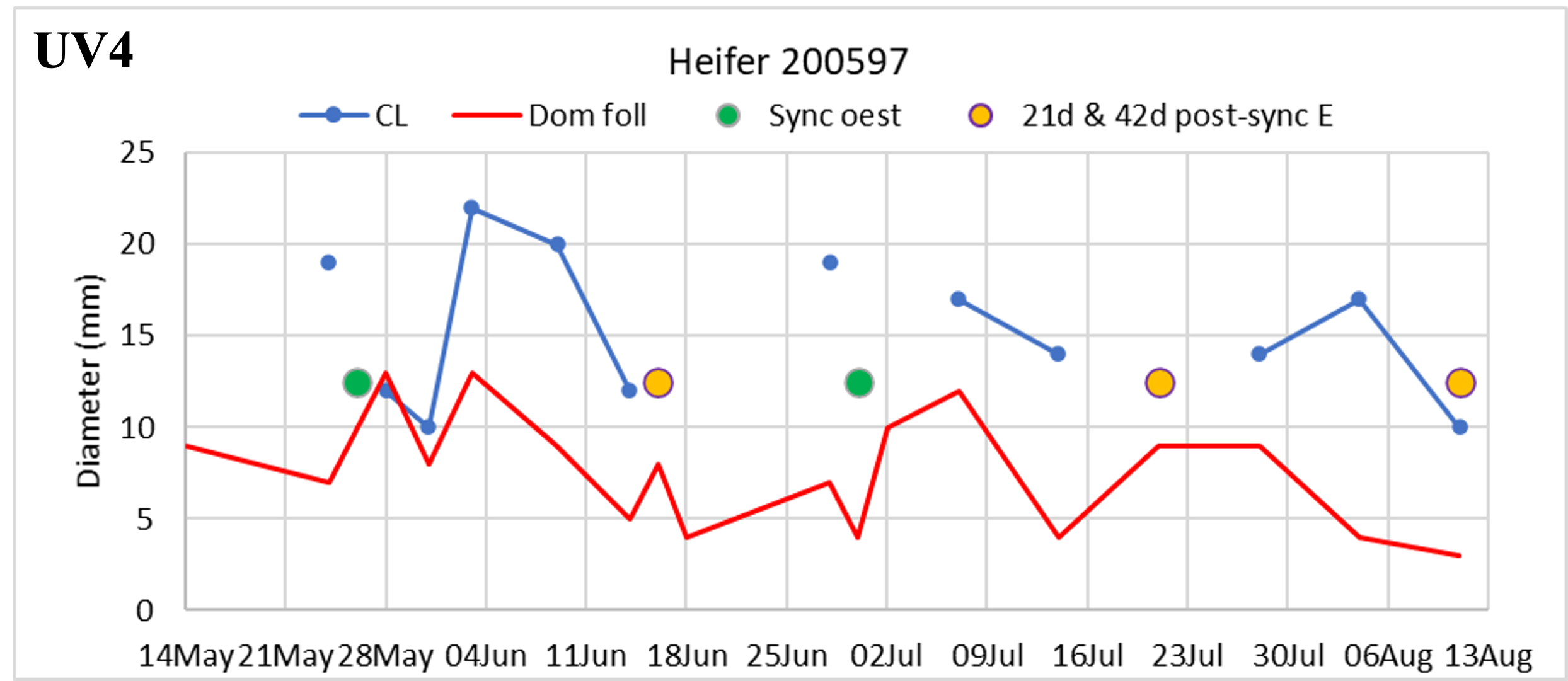 | 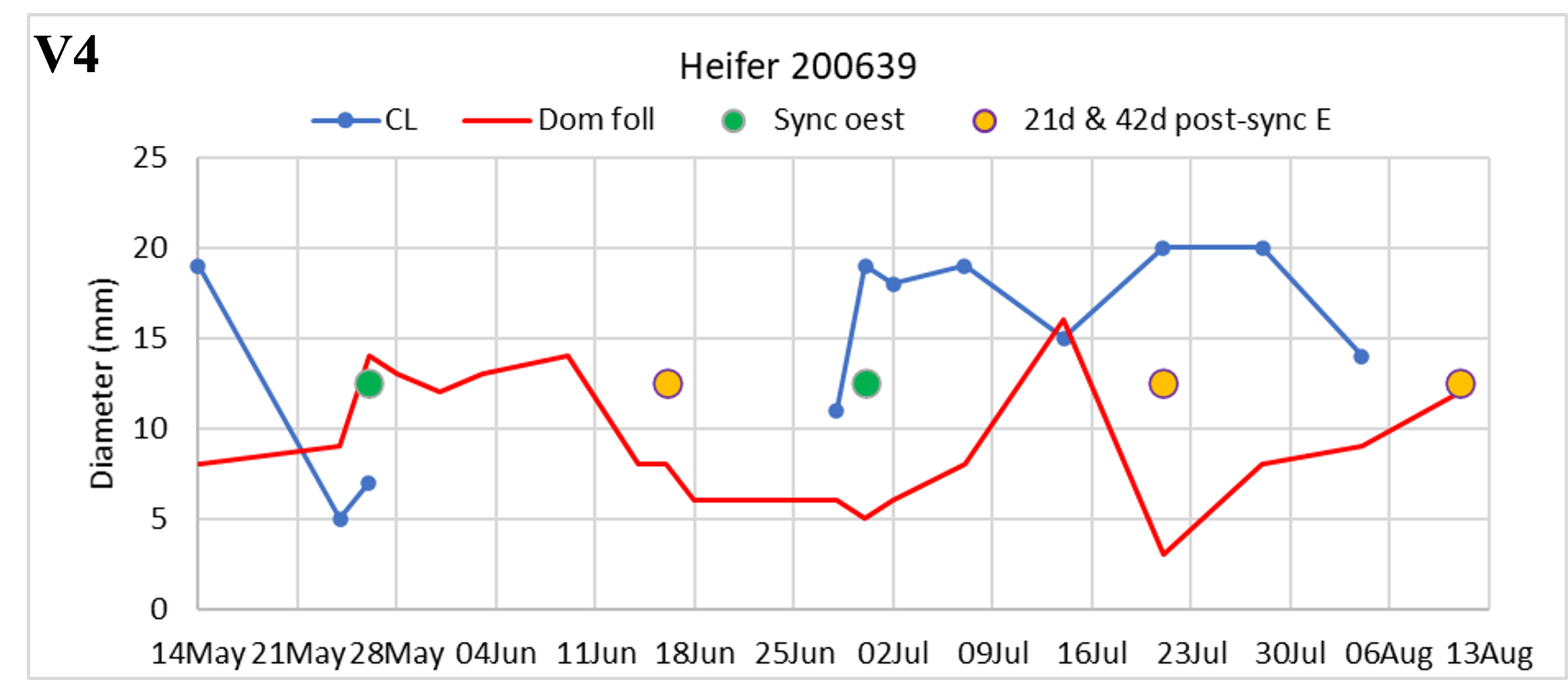 |
| 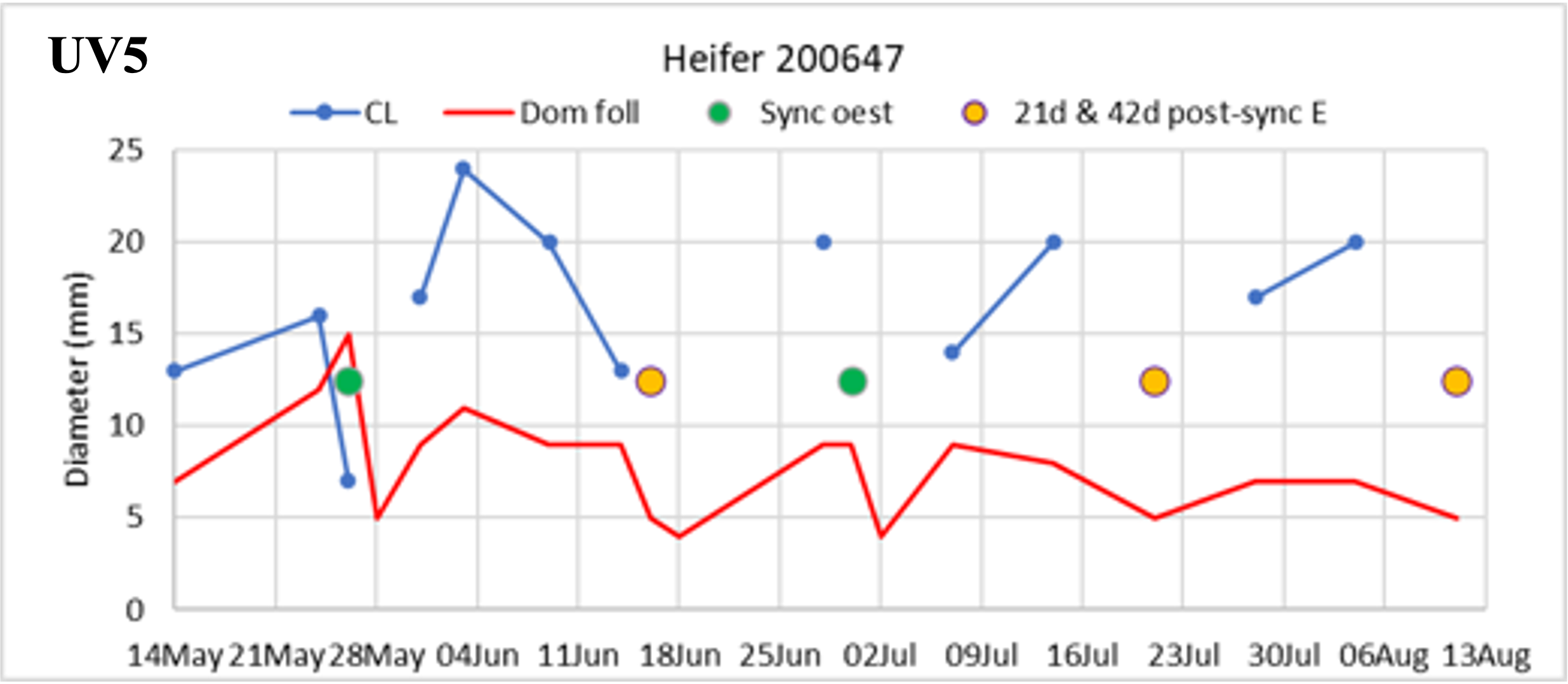 | 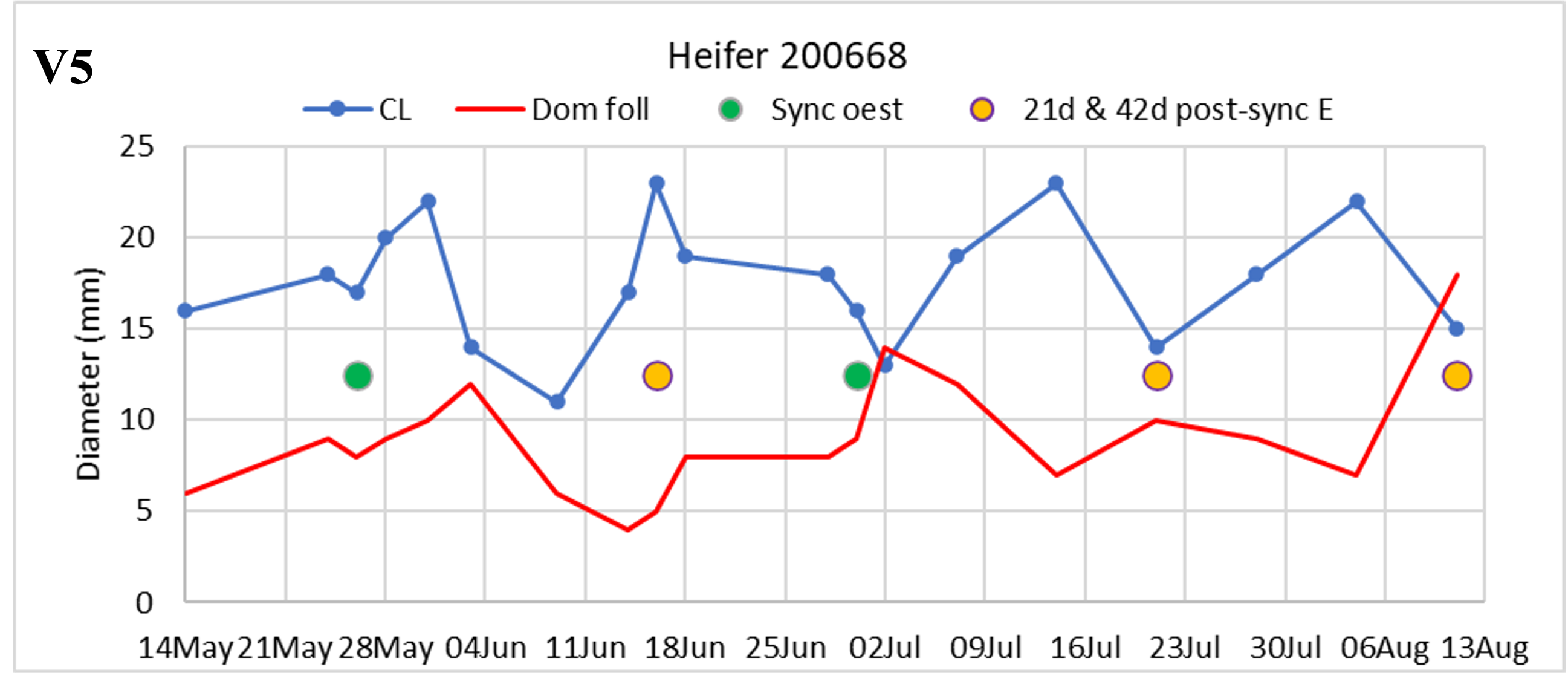 |
| 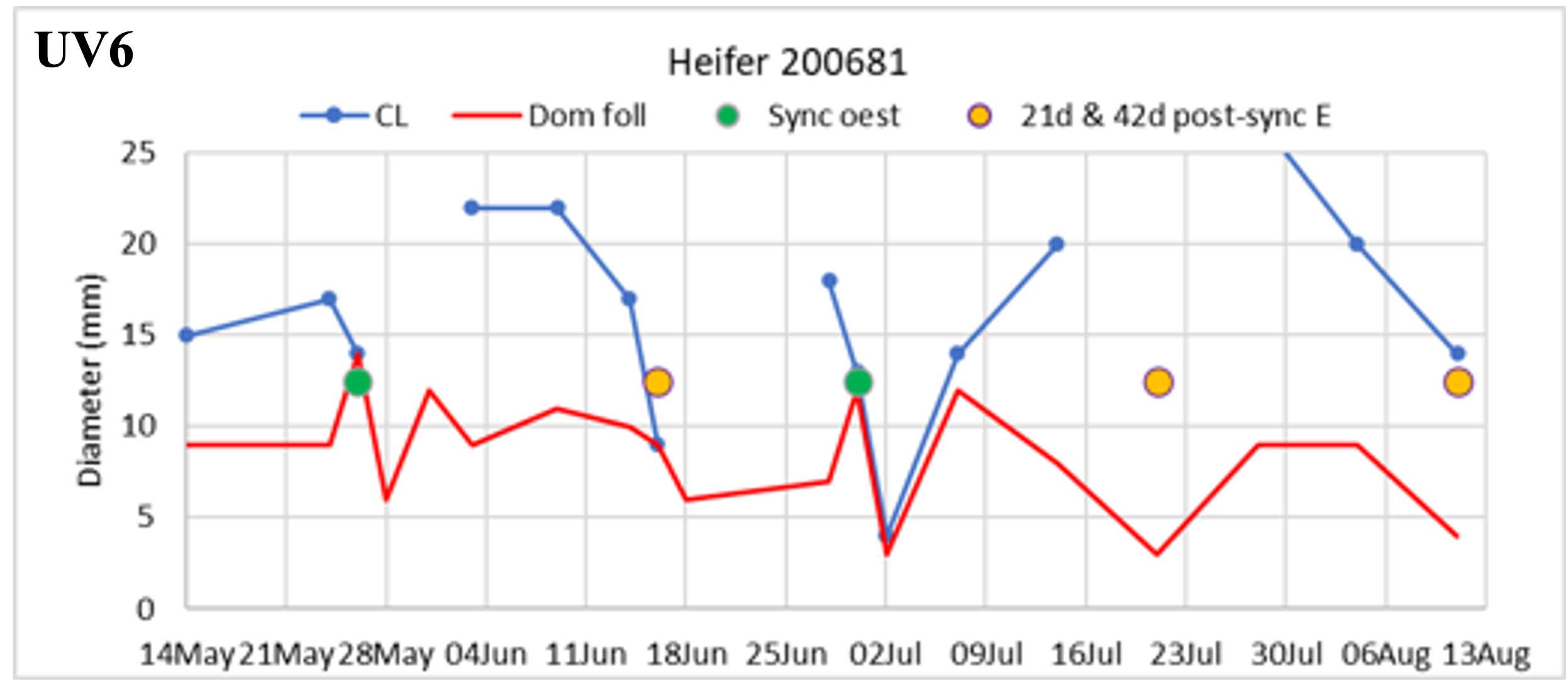 | 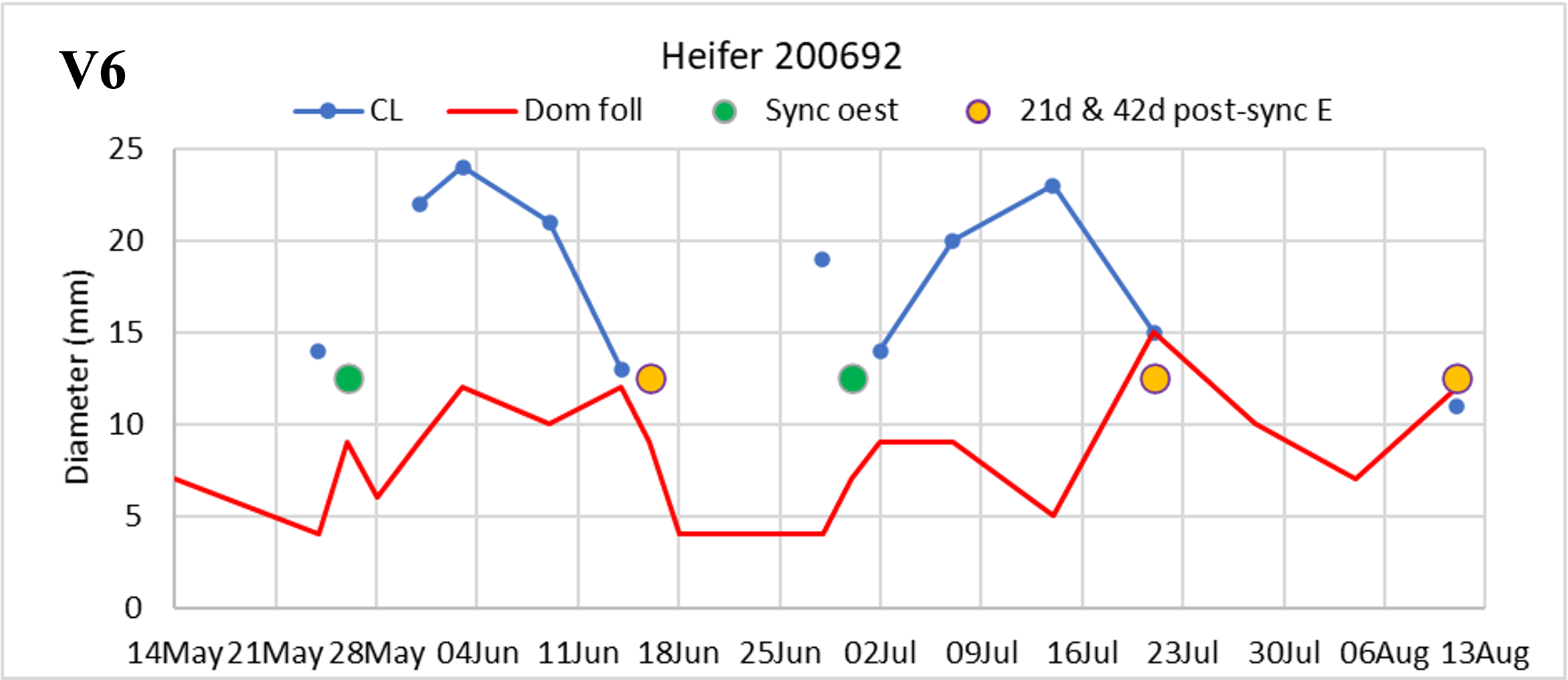 |
| **Figure S1.1.** Real time ovarian ultrasound scanning in individual unvaccinated (UV1-UV6) and vaccinated heifers (V1-V6) prior to each sample collection. The red line dominant follicle (Dom fol); blue dots denote corpus luteum (CL), green dots represent the time of oestrus synchronization (synch oest) and yellow dots- time of oestrus synchronization (21d & 42d post-synch E). One unvaccinated heifer was in anoestrus over the period (200578) and excluded from further analysis. | |

**
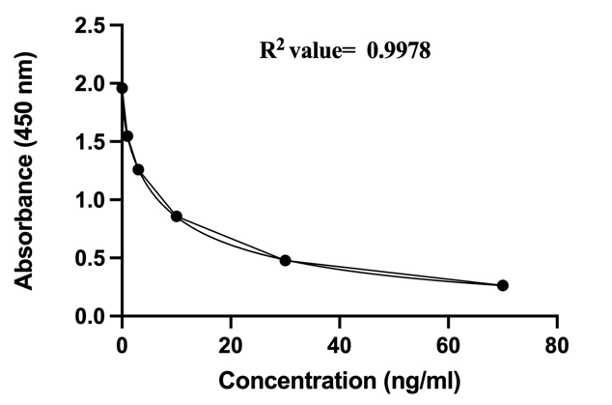
**

**Figure S1.2.** Standard curve generated using four parameter logistic (4-PL) curve fit and progesterone concentrations were detected using linear regression analysis in Graph Pad Prism (version 9.5.1).

**Table S1.1:** Concentration of Progesterone (P4) and absorbance ratio.

| **Date** | **Vaccination status** | **Cycle** | **Concentration (ng/ml)** | **Absorbance (450 nm)** |
| --- | --- | --- | --- | --- |
| **Before challenge** | | | | |
| 28.5.21 | Vaccinated | Oestrus | 0.84 | 1.157 |
| 28.5.21 | Unvaccinated | Oestrus | 0.58 | 1.271 |
| 18.6.21 | Vaccinated | Oestrus | 1.46 | 0.9645 |
| 18.6.21 | Unvaccinated | Oestrus | 0.99 | 1.0985 |
| 217_28.06.21 | Unvaccinated | Dioestrus | 3.79 | 0.6295 |
| 486_28.06.21 | Unvaccinated | Dioestrus | 2.83 | 0.7295 |
| 578_28.06.21 | Unvaccinated | Dioestrus | 2.89 | 0.7225 |
| 597_28.06.21 | Unvaccinated | Dioestrus | 4.54 | 0.57 |
| 647_28.06.21 | Unvaccinated | Dioestrus | 3.69 | 0.6385 |
| 647_28.06.21 | Unvaccinated | Dioestrus | 3.73 | 0.6345 |
| 481_28.06.21 | Vaccinated | Dioestrus | 3.97 | 0.614 |
| 574_28.06.21 | Vaccinated | Dioestrus | 4.49 | 0.574 |
| 594_28.06.21 | Vaccinated | Dioestrus | 3.65 | 0.642 |
| 639_28.06.21 | Vaccinated | Dioestrus | 1.29 | 1.008 |
| 668_28.06.21 | Vaccinated | Dioestrus | 1.24 | 1.0215 |
| 681_28.06.21 | Vaccinated | Dioestrus | 1.78 | 0.893 |
| 217_09.06.21 | Unvaccinated | Dioestrus | 6.28 | 0.47 |
| 486_09.06.21 | Unvaccinated | Dioestrus | 3.38 | 0.668 |
| 578_09.06.21 | Unvaccinated | Dioestrus | 0.59 | 1.269 |
| 597_09.06.21 | Unvaccinated | Dioestrus | 8.31 | 0.3905 |
| 647_09.06.21 | Unvaccinated | Dioestrus | 4.26 | 0.591 |
| 647_09.06.21 | Unvaccinated | Dioestrus | 4.24 | 0.5925 |
| 481_09.06.21 | Vaccinated | Dioestrus | 3.37 | 0.6695 |
| 574_09.06.21 | Vaccinated | Dioestrus | 5.08 | 0.5345 |
| 594_09.06.21 | Vaccinated | Dioestrus | 4.96 | 0.542 |
| 639_09.06.21 | Vaccinated | Dioestrus | 0.80 | 1.1735 |
| 668_09.06.21 | Vaccinated | Dioestrus | 0.00 | 1.887 |
| 681_09.06.21 | Vaccinated | Dioestrus | 0.31 | 1.4455 |
| **After Challenge** | | | | |
| 02.7.21 | Vaccinated | Oestrus | 1.80 | 0.8905 |
| 02.7.21 | Unvaccinated | Oestrus | 0.81 | 1.1675 |
| 21.7.21_vac | Vaccinated | Oestrus | 1.52 | 0.951 |
| 21.7.21_unvac | Unvaccinated | Oestrus | 0.86 | 1.146 |
| 14.7.21 | Vaccinated | Dioestrus | 2.42 | 0.784 |
| 14.7.21 | Unvaccinated | Dioestrus | 3.00 | 0.7095 |
| 04.8.21 | Vaccinated | Dioestrus | 2.42 | 0.784 |
| 04.8.21 | Unvaccinated | Dioestrus | 2.16 | 0.8245 |
